# Metabolomic Study of Sorghum (*Sorghum bicolor*) to Interpret Plant Behavior under Variable Field Conditions in View of Smart Agriculture Applications

**DOI:** 10.1021/acs.jafc.0c06533

**Published:** 2021-01-18

**Authors:** Manuela Mandrone, Ilaria Chiocchio, Lorenzo Barbanti, Paola Tomasi, Massimo Tacchini, Ferruccio Poli

**Affiliations:** † Department of Pharmacy and Biotechnology, University of Bologna, Via Irnerio, 42, 40126 Bologna, Italy; ‡ Department of Agricultural and Food Sciences, University of Bologna, Viale Fanìn 44, 40127 Bologna, Italy; § Department of Life Sciences and Biotechnology (SVeB), University of Ferrara, Piazzale Luciano Chiappini 3, I-44123 Ferrara, Italy

**Keywords:** smart agriculture, NMR metabolomics, quality control, *Sorghum bicolor*, dhurrin

## Abstract

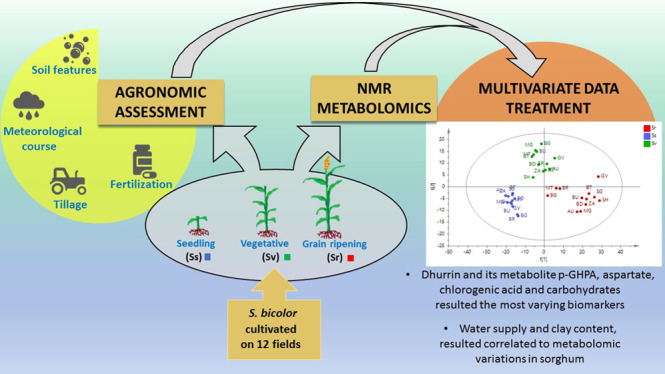

To tackle the urgency of smarter crop management, the complex nature of agricultural ecosystems needs to be better understood, employing and combining different techniques and technologies. In this study, untargeted metabolomics and agro-meteorological survey were coupled to study the variation of *Sorghum bicolor* (L.) Moench metabolome during crop development, in response to environmental and anthropic factors. Twelve crop fields in the Emilia-Romagna region, Italy, were monitored and sampled at different stages, seedling (Ss), advanced vegetative (Sv), and ripening (Sr), and subjected to ^1^H NMR-based metabolomics. The analytical method developed resulted to be successful to quickly analyze different sorghum organs. Dhurrin, a cyanogenic glucoside, resulted to be a biomarker of crop quality and development, and several insights into its turnover and functions were obtained. In particular, *p*-glucosyloxy-2-hydroxyphenylacetic acid was identified, for the first time, as the main metabolite accumulated in sorghum at Sr, after gradual dhurrin neutralization. During plant life, fertilization and biotic and abiotic stress reflected peculiar metabolomic profiles. Water supply and soil features (*i.e.*, clay content) were correlated to metabolomic variations, affecting dhurrin (and related metabolites), amino acids, organic acids, and carbohydrate content. Increase in chlorogenic acid was registered in consequence of predator attacks. Moreover, grain from three fields presented traces of dhurrin and the lowest antioxidant potential, which resulted in poor grain quality. Metabolomics turned out to be a promising tool in view of smart agriculture for monitoring plant growth status and applying appropriate agricultural practices since the early stage of crop development.

## 1 Introduction

Population growth, climate change, and the urgent demand for safe, nutritious, and sufficient food worldwide challenge agriculture to intensify production, while concurrently lowering the food environmental footprint.^1^

To tackle this demand, it is increasingly important to deeply understand the complexity of agricultural ecosystems. In order to achieve this goal, techniques and technologies from many disciplines (biotechnology, agronomy, phytochemistry, microbiology, engineering, and information technology) need to be jointly employed.^2^

The analysis of big data through an inductive (hypothesis-generating) approach, which leads to advances in various fields, is also expected to enable farmers and companies in the agricultural sector to improve their efficiency in a sustainable way.^3^

For instance, considering that most agricultural processes (*i.e.*, crop choice, sowing schedule, growth, and harvest management) depend on the weather, it is of general interest to study how shifts in the weather regimes determine crop variability at regional levels. Thus, advanced and accurate information on weather variables and their quantitative relations to crop processes need to be implemented.^4^ In this framework, untargeted metabolomics coupled with agro-climatic studies could represent a valuable tool to obtain information on the variation of crop metabolome in response to environmental and anthropic factors. Metabolomics, which often relies on untargeted analysis protocols, whose data are handled with multivariate techniques (inductive approach), has already been applied successfully in several areas of research, ranging from human diagnostics and epidemiology to plant sciences.^5^ In the latter field, this approach resulted to be particularly helpful in facilitating the identification of active principles in medicinal plants^6−8^ and for food and botanical quality control, in terms of both nutraceutical/biological properties and fraud detection.^9,10^

This work is focused on *Sorghum bicolor* (L.) Moench, belonging to the Poaceae family and Andropogoneae tribe. Of the large intraspecific variation, we addressed sorghum genotypes suited for grain production, that is, those featuring low plants with large panicles, which are grown as cereals for food and/or feed uses. More specifically, this work focuses on commercial sorghum hybrids producing white (actually pale) grain, that is, whose kernels are devoid of tannins and anthocyanins, and are, therefore, better suited for a vast array of food/feed uses.

The relevant role played by sorghum in global agriculture makes it an important crop to be investigated through metabolomic approaches. Sorghum is the fifth most important cereal in the world; thanks to its good drought resistance, it is intensively cultivated in Africa, Asia, and southwest USA^11^ and is regarded with growing interest in other warm temperate areas of the world. The EU plays a minor role in grain sorghum cultivation: the crop is concentrated in the central Mediterranean areas, with the two major producing countries, France and Italy (approximately 70,000 and 50,000 ha, respectively). However, the cultivation of sorghum for food preparation is recently increasing in Italy and other Mediterranean countries because of its lack of gluten^12^ and its recognized high nutritional value.^13^ Nevertheless, sorghum also produces a cyanogenic glycoside (dhurrin), whose content varies depending on the plant stage and growth conditions,^14^ and because of its toxicity, it must be kept extremely low at harvest.

With these premises, 12 sorghum crop fields in Emilia-Romagna (Italy) were studied from an agronomic and metabolomic viewpoint. In particular, plant samples were harvested at three different stages: seedlings (Ss), advanced vegetative phase (Sv), and grain maturity (Sr), and subjected to ^1^H NMR-based metabolomic analysis. Thus, the relationships among the metabolome, crop parameters, and organs were investigated through multivariate data treatment. The antioxidant activity of the grain was also measured.

On this basis, the present study was aimed to detect the variations of sorghum metabolome associated with growth stage, plant organ, and environmental/crop management factors. Consequently, the NMR-based metabolomic approach was here used also for the broad-spectrum quality control of sorghum grain. The final purpose was to better understand the sorghum metabolome and its response to environment, collecting data potentially useful for implementing smart agriculture practices. Particular attention was given to specific agricultural practices or environmental features, which could positively affect sorghum grain in terms of yield and quality.

## 2 Materials and Methods

### 2.1 Sorghum Fields

**Figure 1:**
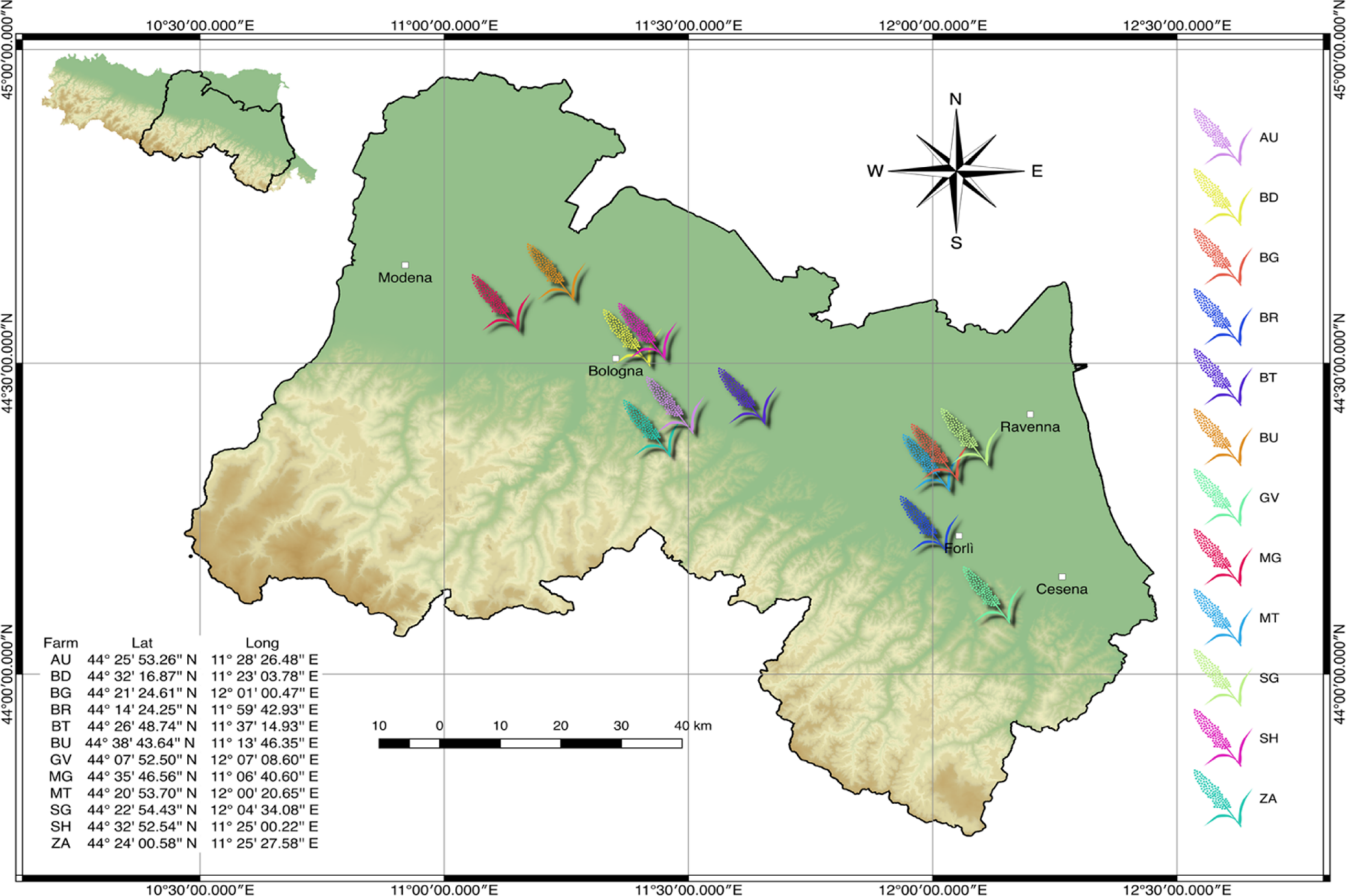
Location of the 12 sorghum fields addressed in this study within the Emilia-Romagna region (Italy). The map of the fields was created in QGIS 2.18.20 (QGIS Development Team, 2016) using the DEM file provided by ISPRA and the shape file provided by ISTAT.

In 2017, a survey was run on commercial sorghum crops in the Emilia-Romagna region, the largest sorghum-producing area of Italy. Twelve fields cultivated with sorghum hybrids producing white grain were selected between 44°07′ and 44°38′ N, and between 11°06′ and 12°07′ E, in the plain area of the region (nine fields; elevation not exceeding 30 m above sea level) or in the footsteps of the Apennine mountains (three fields at an average elevation of 116 m above sea level) (Figure 1). The map of the fields was created in QGIS 2.18.20 (QGIS Development Team, 2016) using the DEM file provided by ISPRA^15^ and the shape file provided by ISTAT.^16^

Sorghum cultivation is fully mechanized in Italy, that is, all operations are carried out with agricultural machinery. Fertilization and weed, pest, and disease control, in most cases, are based on mineral fertilizers and chemical plant protection products. All crop interventions, their timing, and the products used in the 12 fields were annotated, from soil tillage (autumn 2016) to crop maturity (summer 2017). The 12 fields were managed under rain-fed conditions, that is, without irrigation. This is the standard practice for the sorghum crop in the surveyed area.

The course of weather during the crop season (minimum and maximum temperature, precipitation, and relative humidity) was obtained from the regional network of meteorological stations.^17^ Principal soil characteristics such as texture, pH, organic carbon (OC) content at 0–30 cm depth, clay content, silt, and clay content were obtained from specific regional maps.^18^ The shallow water table depth during sorghum growth was obtained from the same online source.^18^

Crop evapotranspiration (ET_C_), representing the evapotranspiration of the specific crop under nonlimiting water supply,^19^ was assessed based on the reference evapotranspiration (ET_0_) calculated with the Hargreaves method.^20^ The relationship between the actual water supply (precipitation) and potential consumption (ET_C_) was investigated as an indicator of potential drought for the crop.

Sorghum in the 12 fields was seeded in the early spring: the average seeding date was April 5, corresponding to 95 ± 5.1 days from the beginning of the year. Sorghum cycle from seeding to harvest lasted an average 127.5 ± 4.9 days after seeding (DAS).

### 2.2 Plant and Soil Sampling

Sorghum samples were collected at three relevant plant stages: seedling (Ss) (26.2 ± 6.1 DAS), booting–initial heading, that is, advanced vegetative stage (Sv) (76.4 ± 5.4 DAS), and grain ripening (Sr) (111.0 ± 5.1 DAS).

At the first sampling (average date, May 1), 40 seedlings were taken from an area of approximately 10 m^2^. Plants were extracted from soil and immediately frozen in liquid nitrogen, then stored at −80 °C until being freeze-dried, and kept in fridge at 4 °C before the analysis.

At the second sampling (average date, June 20), 20 plants were collected from a nearby area and partitioned into stems and leaves. The stems and leaves were immediately frozen in liquid nitrogen and titrated as described for seedlings.

At the third sampling, (average date, July 25), 20 plants were collected from a nearby area and partitioned into stems, leaves, and panicles. The vegetative organs were managed as previously described, whereas the grain from the panicle was dried in a stove at 40 °C, ground, and stored under dark conditions. Additional panicle samples were taken on 0.9 m^2^ crop areas per two replicates, and the grain recovered was weighed and subjected to moisture assessment. Based on these data, the final grain yield per unit crop surface (Mg ha^–1^) at the reference moisture (14%) was determined. The test weight, that is, the apparent volumetric mass (g L^–1^) of the grain, was also determined.

Grain yield and test weight at harvest were subjected to Pearson’s correlations with a series of agro-environmental parameters including tillage depth (TD), organic fertilization, mineral N supply, seeding date, length of the growth period, clay content, OC, ET_C_ – *P*, *P*/ET_C_, and the depth of the shallow water table in July (WTD).

Soil samples at the depth of 0–0.3 m were also collected at the same three times of plant sampling. They were oven-dried (105 °C for 48 h) to determine soil humidity at the beginning (H-ini), middle (H-mid), and end of the sorghum growth season (H-late).

### 2.3 Chemicals

Deuterium oxide (D_2_O, 99.90% D), CD_3_OD (99.80% D), and CDCl_3_ with 0.03% TMS (99.80% D) were purchased from Eurisotop (Cambridge Isotope Laboratories, Inc., France). Standard 3-(trimethylsilyl)-propionic-2,2,3,3-*d*_4_ acid sodium salt (TMSP), sodium phosphate dibasic anhydrous, sodium phosphate monobasic anhydrous, and all the other solvents and chemicals were purchased from Sigma-Aldrich Co. (St. Louis, MO, USA).

### 2.4 Extract Preparation for NMR Analysis

In all cases, samples representative of each field were obtained by pooling material from all the individuals collected on the same field. A 30 mg of freeze-dried and powdered seedlings, leaves, or stem material were extracted using 1 mL of a bland (50:50) CD_3_OD/D_2_O (containing 0.1 M phosphate buffer and 0.01% of TMSP standard). The extracts were sonicated for 30 min and subsequently centrifuged for 20 min at 17,000*g*, and the supernatant (700 μL) was then separated from the pellet and transferred into NMR tubes.

For grain metabolomics, 50 mg of the dried and ground material was subjected to ultrasound-assisted (20 min) extraction in 0.8 mL of CDCl_3_. Then, 0.8 mL of the bland (50:50) CD_3_OD/D_2_O (containing 0.1 M phosphate buffer and 0.01% of TMSP standard) was added, and the samples were subjected to additional sonication for 20 min and subsequently centrifuged for 20 min at 17,000*g*. The obtained supernatant was constituted by a biphasic mixture: 400 μL of the chloroform phase was mixed with 200 μL of CDCl_3_ containing TMS standard (0.03%) and transferred into NMR tubes, whereas 600 μL of aqueous phase was directly transferred into NMR tubes to be analyzed by NMR separately. All metabolomic analyses have been performed during 2017 and 2018.

### 2.5 Dhurrin, *p*-GPHA, and Chlorogenic Acid Prepurification and Structure Elucidation

Freeze-dried leaves at Sr (1.2 g) underwent ultrasound-assisted extraction with 60 mL of CH_3_OH/H_2_O (50:50). The mixture was centrifuged for 20 min at 2469*g*, and then the supernatant was filtered on a Büchner funnel and evaporated using a rotary evaporator under a reduced pressure below 40 °C. The resulting dried extract (200 mg) was dissolved in a proper volume of water and methanol and chromatographed using a C18 column (4 g) by an MPLC instrument (Reveleris, Büchi, Switzerland) equipped with a UV detector and a fraction collector. The extract was eluted with a gradient of H_2_O (solvent A) and MeOH (solvent B) starting from 5% B and reaching 100% B in 25 min, followed by isocratic elution with 100% B for an additional 5 min. The flow rate was 15 mL min^–1^. This resulted in 19 subfractions (from F1 to F19). Dhurrin was obtained in F3 (see the Supporting Information for NMR assignment) and *p*-glucosyloxy-2-hydroxyphenylacetic acid (*p*-GPHA) in F2.

Freeze-dried leaves at Sv (3 g) underwent ultrasound-assisted extraction with 180 mL of CH_3_OH/H_2_O (80:20) two times. The mixture was centrifuged for 20 min at 2469*g*, and then the supernatant was filtered on a Büchner funnel and evaporated using a rotary evaporator under a reduced pressure below 40 °C. The resulting dried extract (700 mg) was dissolved in 50 mL of H_2_O and 50 mL of CHCl_3_; then, the mixture was transferred into a separating funnel to carry out a liquid–liquid partition using 50 mL of CHCl_3_ another two times. The water fractions were combined and dried in the rotary evaporator, yielding 600 mg of crude extract. A 300 mg of this extract was solubilized in a minimum volume of H_2_O and chromatographed using a Sephadex column (180 cm × 2.5 cm internal diameter filled with 210 g of Sephadex LH-20 and 890 mL of CH_3_OH). Methanol was used as the eluent, and the flow rate was 1 mL min^–1^. A total of 17 fractions were collected, and chlorogenic acid was obtained from fraction 10 (see the Supporting Information for NMR and MS data).

*p*-GPHA (D_2_O, 600 MHz): δ 7.39 (d, 2, *J* = 8.6 Hz, H-4, H-8), 7.09 (d, 2, *J* = 8.6 Hz, H-5, H-7), 5.02 (d, 1, *J* = 7.7 Hz, H-1′), 4.89 (s, 1, H-2), 3.88 (dd, 1, *J* = 12.6, 2.3 Hz, H-6′b), 3.71 (dd, 1, *J* = 12.6, 5.9 Hz, H-6′a), 3.56 (qd, 1, *J* = 8.8, 6.2, 2.4 Hz, H-5′), 3.50 (dd, 1, *J* = 8.4, 3.9 Hz, H-2′), 3.47 (t, 1, *J* = 10.3 Hz, H-3′), 3.42 (t, 1, *J* = 9.4 Hz, H-4′); ^13^C NMR (CD_3_OD, 150 MHz): δ 179.4 (CO, C-1), 155.8 (CO, C-6), 134.8 (C, C-3), 128.3 (CH, C-5, C-7), 116.5 (CH, C-4, C-8), 100.5 (CO, C-1′), 76.09 (CH, C-3′), 76.08 (CH, C-5′), 73.3 (CO, C-2), 73.26 (CH, C-2′), 69.72 (CH, C-4′), 66.83 (CH_2_, C-6′a, C-6′b). Positive ESI-MS *m*/*z*: 353 [M + Na]^+^, 369 [M + K]^+^, calculated as 330.29 for C_14_H_18_O_9_. Negative ESI-MS *m*/*z*: 329 [M – H]^−^.

### 2.6 NMR and MS Spectra Measurement

The ^1^H NMR spectra, *J*-resolved, ^1^H–^1^H homonuclear, and inverse-detected ^1^H–^13^C correlation experiments were recorded at 25 °C on a NMR instrument Varian Inova (Milan, Italy), operating at the ^1^H frequency of 600 MHz, equipped with an indirect triple resonance probe. CD_3_OD was used as an internal lock for polar extracts and CDCl_3_ for chloroform extracts. For ^1^H NMR profiling, the relaxation delay was 2.0 s, observed pulse 5.80 μs, number of scans 256, acquisition time 16 min, and spectral width 9595.78 Hz (corresponding to δ 16.0). For the aqueous samples, a presaturation sequence (PRESAT) was used to suppress the residual H_2_O signal at δ 4.83 (power = −6 dB, presaturation delay 2 s). ESI-MS analyses were performed by the direct injection of MeOH solutions of the compounds using a Waters ZQ 4000 (Milford, MA USA) mass spectrometer.

### 2.7 NMR Processing and Multivariate Data Treatment

Free induction decays were Fourier-transformed, and the resulting spectra were phased, baseline-corrected, and calibrated to TMSP at δ 0.0, which was also used as a standard for semiquantitative analysis. Spectral intensities were reduced to the integrated regions of equal width (δ 0.04), corresponding to the region from δ 0.0 to 10.0, with the scaling on standard at δ 0.0 using the NMR MestReNova 12 software (Mestrelab Research, Santiago de Compostela, Spain). The analysis of ^1^H NMR profiles was performed based on an in-house library and comparison with the literature.^21−23^

The regions of δ 5–4.5 and 3.34–3.30 were excluded from the analysis of the aqueous samples because of the residual solvent signals, whereas, for the same reason, the region at δ 7.3 was excluded from the analysis of the chloroform fractions. For multivariate analysis, the models (PCA, PLS-DA, OPLS, and OPLS-DA) were developed using SIMCA-P+ software (v. 15.0, Umetrics, Umeå, Sweden). Data were subjected to Pareto scaling. The supervised models were evaluated by the goodness of fit [*R*^2^*Y* (cum)] and goodness of prediction [*Q*^2^ (cum)], together with the parameters given by the permutation test (performed using 100 permutations).^24^ The coefficients obtained are reported in Table S1 of the Supporting Information. OPLS-DA and PLS-DA were further validated by cross-validated (CV)-ANOVA. For Ss, nine independent OPLS models were built using the altitude, ET_C_ – *P*, organic fertilizer supply, mineral N supply, OC, clay content, silt content, H-ini, and TD in turn as the *y* variable. A total of 10 independent OPLS models were developed for both stems and leaves at Sv, using the abovementioned variables with the addition of H-mid. Lastly, for Sr leaves, stems, and grain, H-late and WTD were also considered.

### 2.8 β-Carotene Bleaching Assay and Statistical Analysis

Sorghum grain samples were obtained by sonication (20 min) of 30 mg of grain powder in 1 mL of MeOH/H_2_O (50:50), followed by centrifugation for 10 min. The supernatants were evaporated in a SpeedVac system (SpeedVac SPD 101b 230, Savant, Italy) to yield the crude extracts. Stock solutions at concentrations of 120, 60, 30, and 15 μg mL^–1^ were prepared. Water was used as the negative control, whereas Trolox stock solutions (from 50 to 500 μM) were used to build the IC_50_ curve of positive control. The assay was developed following the method described by Mandrone *et al.* (2017). The samples were analyzed in triplicate, and the analysis was repeated three times. One-way ANOVA was performed by GraphPad Prism 4 software (La Jolla, CA, USA). Tukey’s post-hoc test was used, and differences at *p* < 0.05 were considered significant.

## 3 Results

### 3.1 Environmental Traits

Soil texture in the 12 fields ranged from being loamy to silty-clayey. The average sand, silt, and clay contents were 23.8 ± 8.3, 46 ± 3.9, and 30.2 ± 8.9%, respectively. The pH was always mildly alkaline (∼7.5), as that of most soils in the area. Organic carbon was quite low (average, 9.5 ± 3.4 g kg^–1^), which is also a typical feature in the surveyed area. Therefore, the soils had a prevailing mineral composition and were supplemented with specific doses of minerals and, sometimes (4 cases out of 12), organic fertilizers to support plant growth.

**Figure 2:**
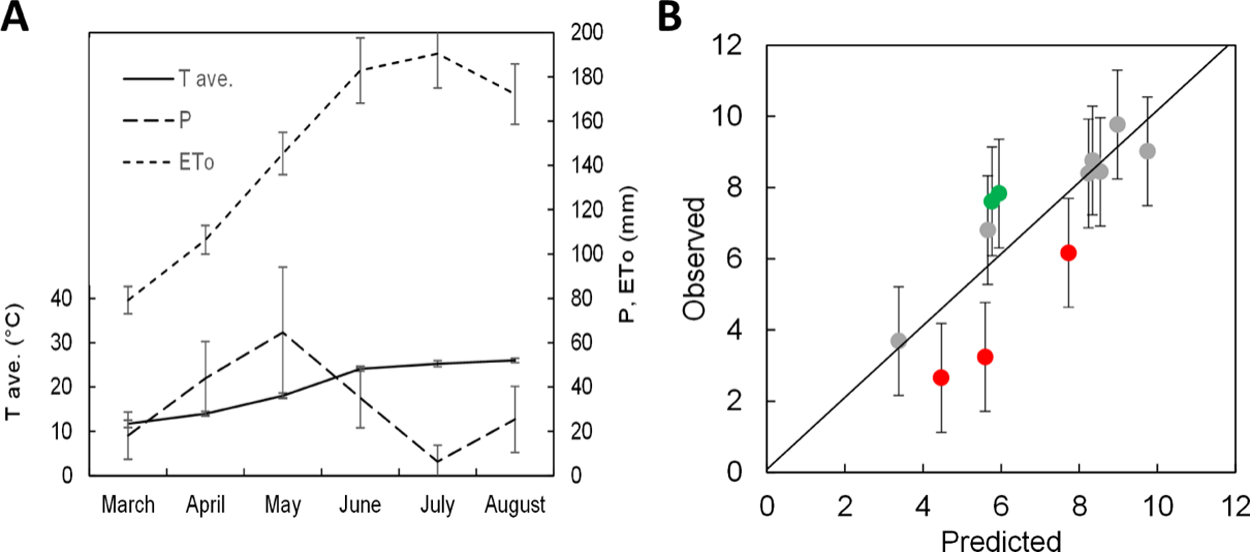
(A) Average temperature (*T*_ave_), precipitation (*P*), and reference evapotranspiration (ET_0_) during the sorghum crop season. Vertical bars indicate ±standard deviation. (B) Predicted grain yield values based on multiple regression involving water table depth and N supply *vs* observed values. Vertical bars indicate ±standard error of the estimate. Data points away from the iso-yield bisecting line by more than the standard error are highlighted in red (predicted > observed) or green (predicted < observed).

In Figure 2A the course of weather during the crop season is represented according to Bagnouls and Gaussen,^25^ that is, plotting for each month the average temperature versus precipitation, the latter in a double scale (right *y* axis) with respect to the former (left *y* axis), and reference evapotranspiration is also included. Based on the cited authors, the months during which double-scaled precipitation falls below the average temperature represent the dry periods of the year. This, in 2017, occurred in June and especially July, that is, during the drought-sensitive reproductive stage. The difference between the natural water supply (*P*) and potential demand from the atmosphere (ET_0_) is even more remarkable. This means that plants grown under a rain-fed regime underwent a severe water deficit, despite good precipitation restoring soil moisture at the beginning of the crop cycle, and the potential contribution from the shallow water table.

### 3.2 Grain Yield and Its Relationship with Agro-Environmental Parameters

Grain yield varied in a wide range from a minimum of below 3 Mg ha^–1^ in a hilly site (GV) to a maximum of almost 10 Mg ha^–1^ in the fertile lowland (MT) (Table 1). The hot and dry weather of the 2017 crop season may have enhanced intrinsic differences among fields. Test weight, which expresses the degree of grain filling during maturation, varied in a much tighter range: from a minimum of 688 g L^–1^ to a maximum of 757 g L^–1^.

**Table 1:** Grain Yield and Test Weight in the 12 Sorghum Fields (Average ± Standard Deviation)

farm	grain yield (Mg ha^–1^)	test weight (g L^–1^)
ZA	3.2 ± 0.2	700 ± 80
AU	6.8 ± 0.5	688 ± 57
BD	8.4 ± 1.2	762 ± 1
SH	8.4 ± 0.1	705 ± 31
MG	7.8 ± 0.9	752 ± 1
BU	8.8 ± 0.1	653 ± 64
BT	9.0 ± 0.7	745 ± 9
MT	9.8 ± 0.5	701 ± 82
BG	7.6 ± 2.2	636 ± 16
SG	6.2 ± 0.2	757 ± 4
GV	2.7 ± 0.2	656 ± 5
BR	3.7 ± 0.6	696 ± 23

The agro-environmental parameters varied remarkably among the 12 fields (Table 2). The fields were all tilled to a relevant depth (0.3–0.45 m). The organic fertilization consisted of livestock manure (BT, MG) or slurry (AU), sometimes in association with a previous lucerne ley (BT, MG). This led to a composite score for organic fertilization ranging from zero, in most cases, to a maximum of 3 in BT. The N supply with mineral fertilizers ranged from a minimum of 92 kg of N ha^–1^ (BR) to a maximum of 230 kg of N ha^–1^ (MT). The length of the growing season from seeding to grain ripening varied in a relatively narrow range (120–135 days), owing to similar thermal course and similar (medium and medium–late) sorghum hybrids being cultivated in all fields. The clay content in the soil varied from a minimum of 19% (MT) to a maximum of 47% (BT). Organic C also varied from a minimum of 0.6% (BT) to a maximum of 1.8% (SG). ET_C_ – *P*, that is, potential water demand (ET_C_) versus supply (*P*), outlined a relevant water deficit in the growth season: from 270 mm (AU) to 402 mm (BR) of seasonal gap between the plant potential uses and actual availability. Another way of expressing the same concept, the ratio of precipitation to crop evapotranspiration (*P*/ET_C_), indicates that precipitation covered from 17% (BT) to 48% (AU) of plant potential uses. This means that more than 50% of potential uses was not covered by precipitation or could only rely on other sources (previous soil water reserve and the shallow water table). Lastly, WTD during the month of July was either below the limit for detection (3 m depth) or between 1.9 m (BT) and 2.67 m (MT and BG).

**Table 2:** Agro-Environmental Parameters in the 12 Crop Fields^a^

farm	tillage depth (m)	organic fert. (scale 0–3)	N supply (kg ha^–1^)	seeding date (DOY)	growth length (d)	clay (% dw)	OC (% dw)	ET_C_ – *P* (mm)	*P*/ET_C_ (%)	WTD (m)
ZA	0.45	0	159	94	135	26	1.0	334	39	>3
AU	0.30	1	161	94	120	35	0.6	270	48	>3
BD	0.30	0	161	96	125	26	0.6	376	33	2.15
SH	0.45	1	170	104	120	33	1.1	363	34	2.15
MG	0.35	2	138	97	125	45	1.2	440	29	2.66
BU	0.35	0	184	96	135	28	1.1	449	27	2.37
BT	0.40	3	184	83	130	47	0.6	449	17	1.90
MT	0.30	0	230	95	125	19	0.9	454	24	2.67
BG	0.35	0	133	96	130	20	1.2	492	21	2.67
SG	0.45	1	161	90	125	25	1.8	485	17	2.32
GV	0.30	1	125	98	130	25	0.7	397	26	>3
BR	0.30	0	92	101	130	33	0.9	402	25	>3

a DOY, day of the year; ET_C_, crop evapotranspiration; *P*, precipitation; OC, organic carbon; WTD, water table depth in July.

The relationships between the agro-environmental and yield parameters are reported in Table 3. Grain yield outlined the significant correlations with the amount of N supplied with mineral fertilizers and WTD in July. Test weight, which is intrinsically less important from a financial viewpoint, was not significantly related to any agro-environmental parameter, nor was it related to yield. Additionally, no significant relationship was observed between the two parameters expressing water deficit (ET_C_ – *P* and *P*/ET_C_) on one side and the two grain attributes on the other side.

**Table 3:** Pearson’s Correlations between Agro-Environmental and Yield Parameters in the 12 Fields^a^

	grain yield	test weight	tillage depth	organic fert.	N supply	seeding date	growth length	clay	OC	ET_C_ – *P*	*P*/ET_C_
test weight	0.24										
tillage depth	–0.01	0.27									
organic fert.	0.19	0.45	0.26								
N supply	0.70	0.17	0.16	0.06							
seeding date	–0.22	–0.36	–0.21	–0.52	–0.38						
growth length	–0.36	–0.38	0.07	–0.24	–0.16	–0.21					
clay	0.15	0.45	0.13	0.82	–0.14	–0.27	–0.16				
OC	–0.03	0.12	0.57	–0.12	–0.08	0.08	0.00	–0.22			
ETC – *P*	0.32	0.02	0.10	0.08	0.10	–0.26	0.30	–0.16	0.50		
*P*/ET_C_	–0.17	–0.10	–0.12	–0.22	–0.02	0.35	–0.31	0.06	–0.36	–0.93	
WTD	0.71	0.49	0.39	0.40	0.46	–0.36	–0.17	0.27	0.08	0.38	–0.41

a *r* ≥ |0.58|, significant at *P* ≤ 0.05; *r* ≥ |0.71|, significant at *P* ≤ 0.01 (*n* = 12).

The multiple stepwise regression of the crop management and environmental factors on grain yield resulted in the following equation
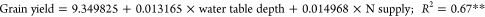


It is perceived from this equation that the two factors singularly best-related with yield (Table 3) were also interacting, contributing to explain a good share (67%) of the total yield variation among the 12 fields.

Based on this equation, predicted grain yield values were plotted versus the values observed (Figure 2B). Seven fields out of the twelve fell near the iso-yield bisecting line, meaning that the predicted and observed grain yield did not diverge significantly. Two fields in the medium–high range (MG and BG) exhibited a higher observed yield than the predicted yield, implying a better crop husbandry by the farmer or some unidentified factor determining this result. Lastly, three fields outlined lower observed values than predicted values (ZA, SG, and GV): the field in the medium yield range (SG) (observed and predicted of 6.2 and 7.7 Mg ha^–1^, respectively) suffered a severe hailstorm at the beginning of the reproductive stage, which can explain the result; the other two cases in the low yield range (ZA and GV) featured a poor crop husbandry or some unidentified factor for this outcome.

### 3.3 Metabolomic Analysis of Leaves and Stems

#### 3.3.1 Seedling Analysis

**Figure 3:**
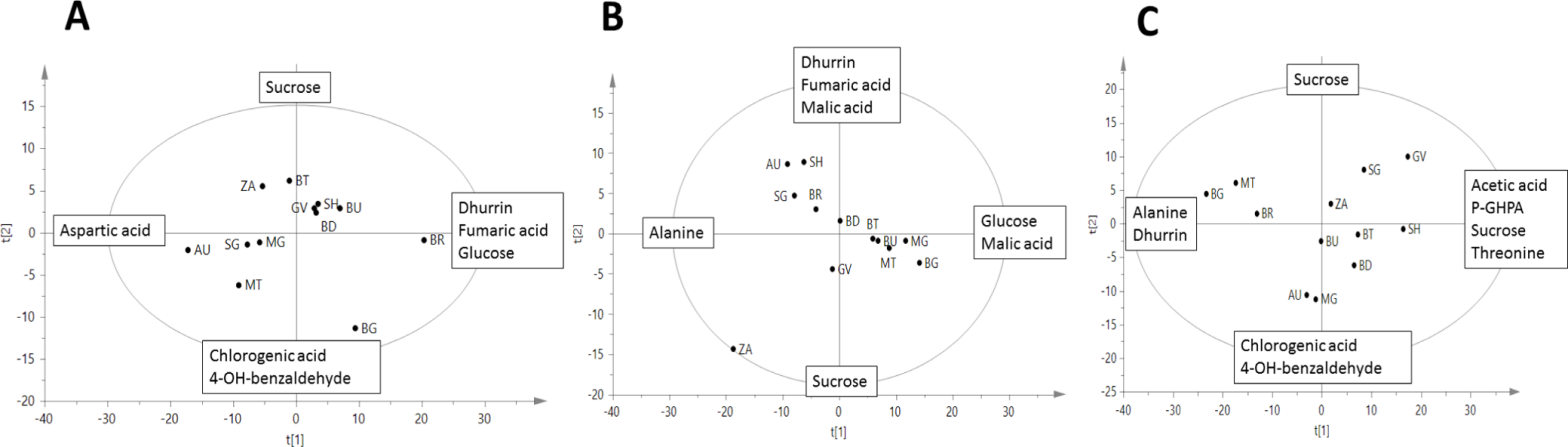
^1^H NMR-based PCA score scatterplot of sorghum at three different growth stages: (A) Ss (seedling stage)––dhurrin, glucose, and fumaric acid increase along the positive side of component *t*[1], whereas aspartic acid follows an inverted trend, increasing on the negative side of *t*[1]. Sucrose increases along the positive side of component *t*[2], whereas *p*-HBA and chlorogenic acid increase on the negative side of *t*[2]; (B) leaves at Sv (advanced vegetative stage)––dhurrin and fumaric acid increase on the positive side of *t*[2] with a trend opposite to that of sucrose; glucose increases on the positive side of *t*[1] with a trend opposite to that of alanine; malic acid increases on the positive side of both *t*[1] and *t*[2]; (C) leaves at Sr (grain ripening)––*p*-GHPA, sucrose, acetic acid, and threonine increase along the positive side of component *t*[1], whereas alanine and dhurrin follow an inverted trend, increasing on the negative side of *t*[1]. Glucose increases along the positive side of component *t*[2], whereas *p*-HBA and chlorogenic acid increase on the negative side of *t*[2].

**Figure 4:**
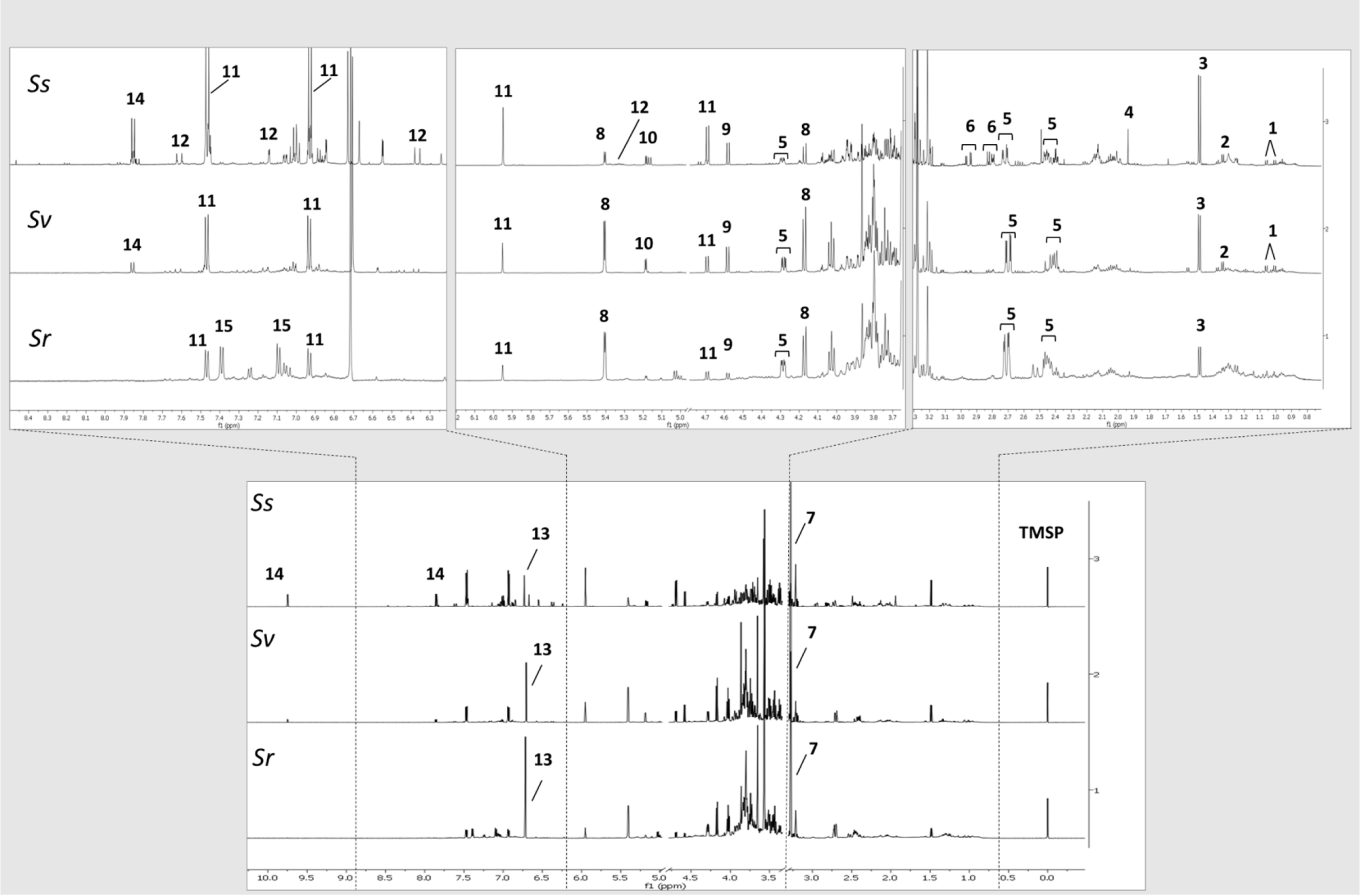
^1^H NMR full spectra of representative samples of sorghum leaves (collected on the SG field) at Ss, Sv, and Sr. Residual solvent signals have been removed; TMSP = standard. Numbers indicate the diagnostic signals of the most variated metabolites: 1 = valine, 2 = threonine, 3 = alanine, 4 = acetate, 5 = malic acid, 6 = aspartic acid, 7 = glycine betaine, 8 = sucrose, 9 = β-glucose, 10 = α-glucose, 11 = dhurrin, 12 = chlorogenic acid, 13 = fumaric acid, 14 = *p*-HBA, and 15 = *p*-GHPA.

The untargeted ^1^H NMR-based metabolomic analysis pointed out the differentiation of sorghum growing on diverse fields (Figure 3). The diagnostic signals of all identified metabolites are reported in Table S2, and the exemplificative spectra are shown in Figure 4.

According to the PCA performed on samples at Ss (Figure 3A), dhurrin, 4-hydroxy-benzhaldeyde (*p*-HBA), and chlorogenic acid were identified as the most varying secondary metabolites, whereas, among the primary metabolites, glucose, sucrose, fumaric acid, and aspartic acid were the most relevant for sample discrimination (loading plots of the models are given in Figures S1–S3). From NMR profiling, it is observed that dhurrin resulted to be the most abundant secondary metabolite produced at Ss, with the concentration ranging from 35 to 101 mg g^–1^ of dried weight (DW).

BG and BR exhibited a peculiarly diverse metabolomic profile, both being characterized by a higher amount of dhurrin compared to the other samples. Furthermore, BR also showed increased glucose and fumaric acid, whereas BG was particularly enriched in chlorogenic acid and *p*-HBA. Moreover, the PCA revealed an inverted trend between aspartic acid and dhurrin concentration in plants at Ss.

#### 3.3.2 Late Vegetative Stage Analysis

Leaves and stems at Sv were first analyzed separately by building two independent PCA models. Dhurrin was found in both stems and leaves, and in this stage, another aromatic compound was visible in the spectra. It was then isolated and identified (by NMR and MS analysis) as *p*-GPHA. Moreover, at this stage, in contrast to Ss, aspartate did not significantly contribute to the diversification among the leaf samples, and it was no more correlated to the decrease in dhurrin concentration.

With regard to leaves at Sv, sucrose content also resulted to be an important factor of discrimination among samples, together with aliphatic amino acids, fumaric acid, and dhurrin (Figure 3B). The model placed ZA as an outlier because of its high content of chlorogenic acid, *p*-GPHA, and acetate.

One of the most varying metabolites in the stems at Sv resulted to be glucose, increasing linearly with aspartate, aliphatic amino acids, and dhurrin (Figure S4A), while showing a trend opposite to that of chlorogenic acid. This latter metabolite resulted to be peculiarly abundant also in ZA stems.

In order to deepen the metabolomic differences among stems and leaves at Sv, a supervised model OPLS-DA was developed (Figure S4B), classifying samples according to plant organs. This analysis highlighted that stems at Sv were generally characterized by a higher concentration of primary metabolites, except for alanine and fumaric acid. On the other hand, leaves at Sv resulted to be more enriched in secondary metabolites, especially dhurrin. Generally, the metabolite trends observed for the leaves and stems at Sv were not overlapping, with some exceptions: SH, which expressed a high level of dhurrin in both leaves and stems at Sv; GV, which showed a high content of sucrose in both leaves and stems; and ZA, characterized by the accumulation of chlorogenic acid in both organs.

#### 3.3.3 Ripeness Stage Analysis

Different metabolomic profiles were also observed for sorghum leaves at Sr (Figure 3C). Once again, dhurrin ranked among the most varied metabolites, as it followed an inverted trend versus *p*-GPHA, whereas aspartic acid was no more detectable in the leaves at this stage.

BG, MT, and BR leaves at Sr were found to be more similar in their metabolome, characterized by higher amounts of dhurrin and alanine, in contrast to GV and SH, where these two compounds were strongly decreased in spite of increased *p*-GPHA, threonine, and sucrose. Lastly, AU and MG showed an increment in *p*-HBA and chlorogenic acid.

For the stems at Sr, the most varying metabolites were acetic acid, fatty acids, carbohydrates (glucose and sucrose), malic acid, and glycine betaine (Figure S4C).

As observed at Sv, the metabolome of stems and leaves did not follow the same variation trend. Noteworthily, BG leaves were the poorest in terms of sucrose content, whereas its stems were among the richest.

The differences between leaves and stems at Sr were more deeply investigated through the OPLS-DA model (Figure S4D). This analysis clearly showed that the leaves at Sr have a higher content of the main metabolites except for malate and fumarate, which are more concentrated in the stems.

### 3.4 Assessment of Metabolomic Variation during Plant Development

In order to highlight the variations in the metabolome of different organs during sorghum ontogeny, all data previously obtained on leaves at different stages were summarized in another PLS-DA model, where the given classes were the three stages of harvesting. Similarly, an OPLS-DA model was built to compare the stems at Sv and Sr.

**Figure 5:**
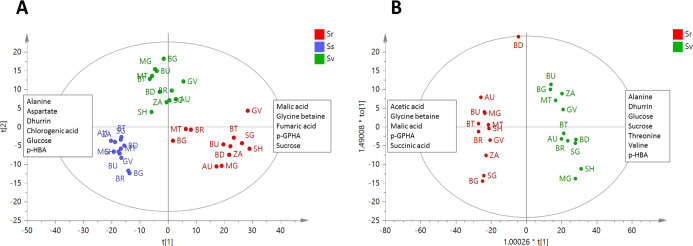
^1^H NMR-based (A) PLS-DA score scatterplot comparing Ss and leaves at Sv and Sr. Leaves of MT, BR, and BG are more closely related between the two stages, in particular for their concentration of dhurrin which increases along the negative side of component *t*[1]. From the permutation test, *R*^2^ = 0.916; *Q*^2^ = 0.893; *p* (CV-ANOVA) = 1.26 × 10^–23^; *F* (CV-ANOVA) = 40.19. (B) OPLS-DA of stems at Sv and Sr; BD presents a peculiar profile because of the high concentration of an unknown aromatic compound. From the permutation test, *R*^2^ = 0.94; *Q*^2^ = 0.898; *p* (CV-ANOVA) = 3.75 × 10^–9^; *F* (CV-ANOVA) = 41.67.

The PLS-DA score scatterplot (Figure 5A) highlighted the peculiar variations, which occurred in leaf metabolome during sorghum growth. In particular, dhurrin, *p*-HBA, chlorogenic acid, glucose, alanine, threonine, and aspartate content decreased progressively from the seedling to the ripening stage. On the other hand, *p*-GPHA, glycine betaine, fumaric acid, sucrose, and malic acid followed the opposite trend, reaching the highest concentration in the leaves at Sr. Based on this, the developed model pointed out that BG, MG, and MT leaves at Sr possessed a metabolome closer to the leaves at Sv.

Variable influence on projection (VIP) values of the model were also investigated. Variables having VIP values over 1.0 are generally considered as the most significant in terms of contribution to group separation. Accordingly, in the developed PLS-DA model, metabolites with a VIP cutoff value over 1.0 were: glycine betaine (2.92), sucrose (2.44), dhurrin (2.24), malic acid (2.08), chlorogenic acid (1.91), *p*-GPHA (1.56), *p*-HBA (1.46), and threonine (1.16), as listed in Table S3.

The OPLS-DA model (Figure 5B) built to compare stems at Sv and Sr showed, as in the case of leaves, that dhurrin and *p*-HBA tend to decrease during plant life, with the consequent increase of *p*-GPHA. Moreover, stems at Sv showed the highest content of aliphatic amino acids (alanine, valine, and threonine) and sugars (glucose and sucrose). On the other hand, stems at Sr exhibited an increased amount of organic acids (malate, succinate, and acetate) and glycine betaine.

In this case, metabolites with a VIP cutoff value over 1.0 were: glycine betaine (2.28), glucose (2.07), and sucrose (1.67), as listed in Table S3.

This model also stressed the peculiarity of BD stems at Sr because of the presence of an unknown aromatic compound (spectral signals at δ 7.9, 8.2, and 8.4). Moreover, it showed a low content of succinate, similar to the stems of plants at Sv.

### 3.5 Grain Metabolomics and Antioxidant Properties

Grain collected from the 12 fields were analyzed using a biphasic protocol of extraction, which led to the obtainment of two fractions analyzed separately. As the metabolite content of grain is quite low compared to the content in leaves and stems, this extraction procedure was required in order to concentrate grain metabolites (especially secondary metabolites such as dhurrin), making them detectable and quantifiable by ^1^H NMR.

No differences were found among the metabolomes of the lipophilic fractions (data not shown), which appeared to be mainly constituted of fatty acids, whereas the hydrophilic extracts contained the metabolites of interest and were differing among each other.

**Figure 6:**
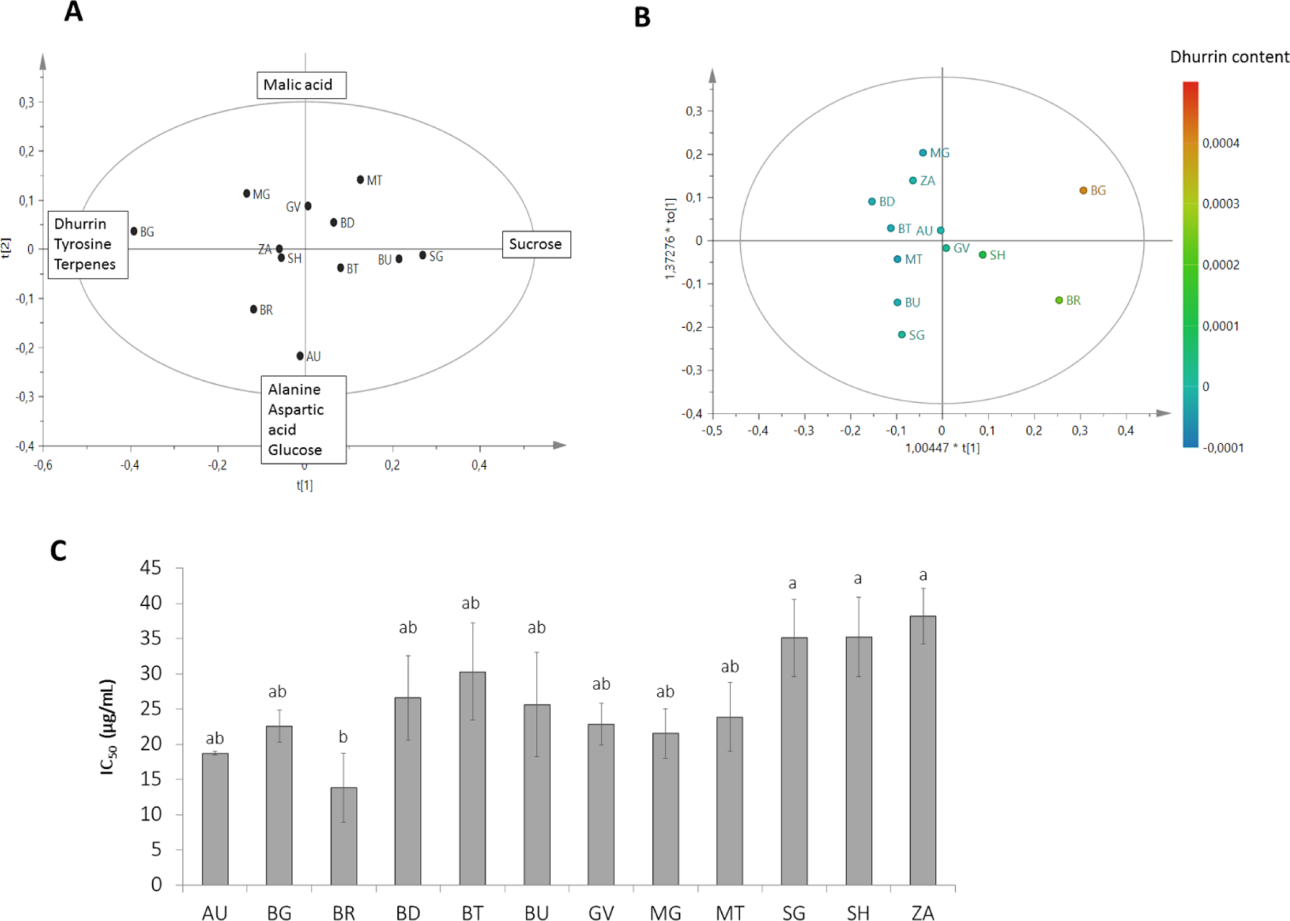
Analysis of sorghum grain. (A) ^1^H NMR-based metabolomic PCA score scatterplot showing sucrose increasing along the positive side of component *t*[1], whereas dhurrin, tyrosine, and terpenes follow an inverted trend, increasing on the negative side of *t*[1]. Malic acid increases along the positive side of component *t*[2]; alanine, aspartic acid, and glucose increase on the negative side of *t*[2]. (B) OPLS based on the intensity of dhurrin diagnostic signal (at δ 5.95) used as *y* variable. (C) Antioxidant activity based on BCB test and subjected to ANOVA test; different letters indicate significantly different values at *p* < 0.05.

Dhurrin traces were still found in some samples (ranging from 10 to 76 μg g^–1^ DW). As shown by PCA (Figure 6A), grain of BG was the most enriched in dhurrin, together with *p*-GPHA. Moreover, SG grain showed the highest amount of sucrose, MT the highest amount of malic acid, whereas BR and AU showed the highest amounts of alanine, aspartic acid, and glucose (loading plots of the model are given in Figure S6).

Dietary exposure to elevated levels of some cyanogenic glycosides in the edible parts of crop plants has the potential to cause acute cyanide poisoning, constituting a health risk for humans and domestic animals.^14,26^ Owing to the fact that the presence of dhurrin compromises grain quality, in order to highlight the occurrence of this metabolite in the sorghum grain, an OPLS model was built (Figure 6B) using dhurrin concentration as the *y* variable, which is represented by the intensity of the spectral bin at δ 5.91–5.95, referring to dhurrin diagnostic proton (H-2) in the α position in the nitrile group.

This model allowed us to better visualize that BG, BR, and SH grain still presented traces of dhurrin, whereas the other fields presented no dhurrin at all, resulting in better grain quality.

However, among the better quality samples, there were still some differences, and they could be divided into three groups: one composed by MG and ZA; another by MT, BD, MT, AU, and BT; and the last one by BU and SG. The last group contained high amounts of sucrose but lower contents of all the other metabolites. The group comprising MG and ZA was characterized by more lipids and aliphatic amino acids such as valine. The group composed by all the remaining fields presented high glucose and malic acid, trigonelline, and some aromatic compounds.

β-carotene bleaching (BCB) assay was performed to assess the grain antioxidant properties. All grain, especially BR, were found to actively inhibit the *in vitro* peroxidation of lipids; the statistical analysis highlighted a lower antioxidant potential for SH, SG, and ZA, with no significant differences among them (Figure 6C).

### 3.6 Correlation between Agro-Environmental Parameters and Metabolomic Profiles

Different supervised models (OPLS) were built to establish significant correlations between the variations in sorghum metabolome and the monitored agro-environmental parameters, which were used in turn as *y* variables of the related model, where *x* variables were again the bucketed signals of ^1^H NMR spectra.

**Figure 7:**
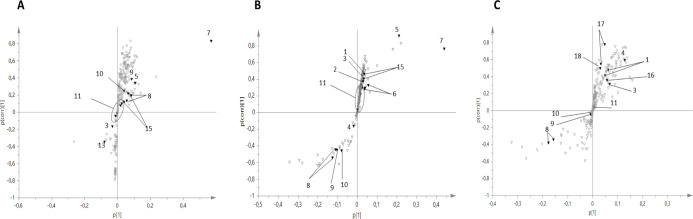
*S*-Plot obtained from different OPLS models using clay as the *y* variable. (A) Effect of clay (Cls) variations on the metabolome of Sr leaves, (B) Sr stems, (C) Sr grain: 1 = valine, 2 = threonine, 3 = alanine, 4 = acetate, 5 = malic acid, 6 = aspartic acid, 7 = glycine betaine, 8 = sucrose, 9 = β-glucose, 10 = α-glucose, 11 = dhurrin, 12 = chlorogenic acid, 13 = fumaric acid, 14 = *p*-HBA, 15 = *p*-GHPA, 16 = lipids, 17 = aromatics, and 18 = trigonelline.

The *S*-plot, associated to a specific OPLS model, indicated which specific metabolites varied in correlation to the surveyed agro-environmental variable used as *y* (Figures 7 and S6).

For Ss and Sv leaves, no correlation could be established among variations in the metabolome and the surveyed agro-environmental parameters. Conversely, specific soil features (silt content, H-ini, and OC) were found to be correlated to the variation of Sv stem metabolome. In particular, OC was directly related to the glucose, glycine betaine, aspartate, valine, and threonine content of Sv stems. Sucrose content decreased at raising OC and increased at raising silt content. This latter variable, in turn, showed an inverted relation with acetate and dhurrin. A general increase in the whole metabolome was associated to the highest levels of H-ini, with a remarkable increment of sucrose, glucose, and aliphatic amino acids (aspartate, valine, and threonine).

The only parameter found to be correlated to the stems at Sr was the clay content, whereas for the leaves at Sr, together with clay, water deficit (ET_C_ – *P*) and TD were also correlated to the metabolomic variations. In the case of Sr stems, malic acid, fumaric acid, glycine betaine, *p*-GPHA, alanine, aspartic acid, and threonine increased together with clay, whereas sucrose and glucose were inversely related to this parameter. For leaves at Sr, increased clay and TD were generally associated with the increase of all metabolites, most prominently sucrose. The highest variation of dhurrin and alanine in Sr leaves was associated to ET_C_ – *P* with an inverted trend to glucose, malic acid, and *p*-GPHA.

With regard grain, the models were built after excluding BG, which was clearly too different from the other samples, probably because of delayed ripening. The grain metabolome resulted to be influenced by ET_C_ – *P*, WTD, and clay, whereas no correlation was established with the panicle weight and grain yield.

ET_C_ – *P* resulted to be negatively correlated with alanine, glucose, valine, and acetate, whereas it scarcely affected sucrose. WTD was positively correlated with sucrose and aromatics (δ 6.59–6.67), possibly flavonoids, and negatively correlated with glucose, alanine, and valine. Clay content was negatively correlated with sucrose and malic acid, whereas it positively correlated with aromatics, alanine, valine, and acetate.

The analyzed parameters were not strictly related to dhurrin content, even though this compound resulted to be little increased under increasing ET_C_ – *P*.

## 4 Discussion

The metabolomic analysis allowed us to identify the most prominent metabolites in diverse sorghum organs, their variation during plant growth, and the diversification of plant metabolome under different crop conditions.

**Figure 8:**
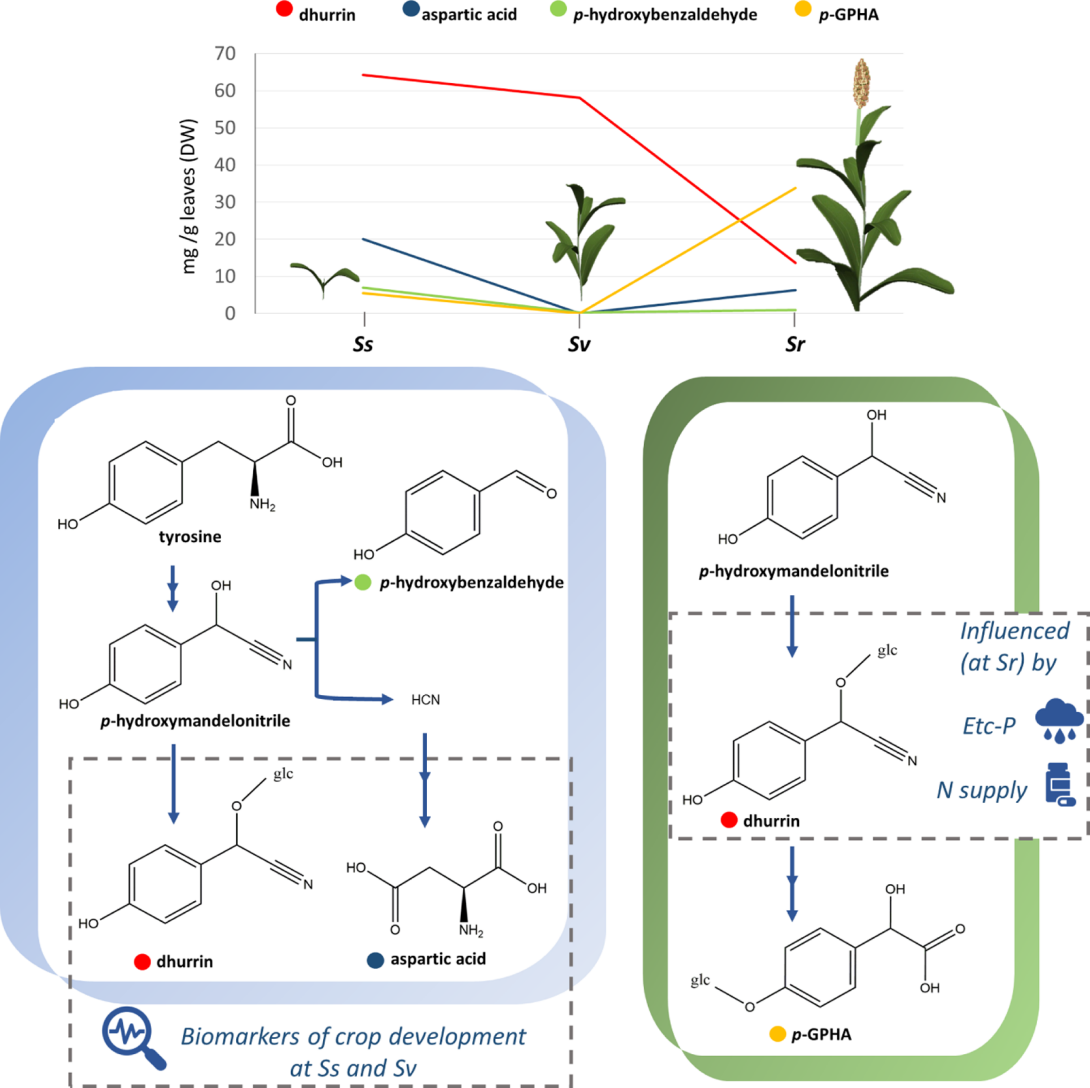
Schematic representation of dhurrin and related metabolite pathways and variations (in leaves) during plant development. The variation trend reflects in different catabolic pathways underwent by dhurrin. On this basis, the levels of dhurrin and aspartic acid in Ss and Sv are the biomarkers of crop development. At Sr, dhurrin content resulted to be affected by overfertilization and water depletion.

Dhurrin is one of the most peculiar secondary metabolites produced by sorghum, and the results of this work provide new insights into its biological role, the variations of its content during plant development, as well as some aspects of its turnover pathway (Figure 8).

Dhurrin is a cyanogenic glycoside, a class of metabolites that, upon tissue disruption, are hydrolyzed by endogenous β-glucosidases into cyanohydrin aglycone, which in turn releases the toxic hydrogen cyanide (HCN).^27^

In this work, dhurrin was found to be particularly abundant at Ss. This is consistent with its defensive role as a deterrent against insects and generalist herbivores,^28,29^ a function particularly important during the early stage of plant life. On the other hand, dhurrin, as other cyanogenic glycosides, may serve additional functions, such as resistance against abiotic stresses and nitrogen storage/buffer.^30,31^ The latter role might be important, especially during ripening processes; in fact, assimilation of nitrogen from the soil in cereals is often insufficient to supply the developing grain; thus, a complementary nutrient is made available through dhurrin remobilization from the leaf tissue.^30^

Moreover, according to the analysis performed on plants at Ss, it is observed that when aspartic acid increases, dhurrin decreases. These data support the dhurrin turnover pathway proposed by previous studies,^32,33^ according to which dhurrin is hydrolyzed to form the intermediate *p*-hydroxymandelonitrile (*p*-HMN), which is then converted into *p*-HBA and HCN. As a mechanism of detoxification, the released HCN is incorporated into β-cyanoalanine, to be finally converted into asparagine and aspartic acid, with a concomitant release of ammonia. Thus, according to the results of our study, when the dhurrin concentration decreases, aspartate production increases. Nevertheless, this trend was observed at Ss, whereas at Sr, a lowered dhurrin content in all organs (leaves, stems, and and grain) was associated to the accumulation of *p*-GPHA, instead of aspartic acid. This latter amino acid was not even found in Sr leaves, stems, and grain. Moreover, in this stage, *p*-HBA was also no more detected.

To the best of our knowledge, this is the first report of *p*-GPHA occurrence in sorghum and of its accumulation as the main metabolite after dhurrin catabolism. In contrast to Ss, when dhurrin was converted into aspartate and HCN was neutralized in NH_3_, at Sr, dhurrin underwent a different turnover pathway, probably avoiding the release of HCN. Nielsen *et al.*(30) proposed two putative dhurrin turnover pathways in sorghum grain, one culminating in the production of dhurrin acid and the other in *p*-glycosyloxy-phenylacetic acid (*p*-GPA). According to our results, none of them is the most prominent final metabolite of dhurrin pathways in sorghum at Sr, whereas the metabolomic analysis suggested *p*-GPHA as the main metabolite resulting from dhurrin turnover. Considering the structure similarity of *p*-GPHA, dhurrin acid, and *p*-GPA, the latter two could be the intermediates from which *p*-GPHA is finally obtained.

Besides the aspects related to dhurrin catabolism, the overall metabolomic picture obtained from the seedling analysis suggested three different trends among the 12 sorghum fields. Four out of them showed the lowest content of dhurrin and *p*-HBA (with the resulting glutamate increase). These crop fields were possibly turning into a different development stage, characterized by a decrease of sucrose and glucose, which were likely used as carbon sources for the growth. An opposite trend was observed for two crop fields (BG and BR), which were characterized by the highest concentration of dhurrin. Interestingly, this peculiar metabolomic profile showed at Ss by BG and BR was associated to a delayed trend of development, which became evident at the final stage of the survey (Sr).

However, BG seedlings showed a very peculiar profile, diverging from BR. In fact, BG was also characterized by an extremely high concentration of *p*-HBA and chlorogenic acid. These metabolomic features could not only be related to delayed plant development but also to a reaction against predators. In fact, if *p*-HMN is converted into *p*-HBA, instead of dhurrin, HCN is consequently released as an eventual defensive strategy.^34^ The increment of chlorogenic acid is an additional evidence of a predator response, as this compound is already known to be involved in plant defense.^35^

The other six fields at Ss were characterized by sucrose accumulation, which might indicate a more quiescent metabolic activity, characterized by sucrose storage. During Sv, when dhurrin and *p*-HBA decreased, an increment of sucrose was once more registered. In this stage, BG and GV leaves were differing from the others for their increased sugar content and decrease of all other metabolites. Specifically, in GV, both glucose and sucrose increased, whereas in BG, only sucrose was highly concentrated. The highest content of dhurrin was found in SH leaves, whereas ZA leaves revealed a peculiar profile, with a high concentration of chlorogenic acid. In this stage, aphids were visible on ZA leaves, corroborating chlorogenic acid as defense against predator attack.

The progressive decrease of dhurrin during plant development was clearly evidenced by the PLS-DA model, comparing leaves at Ss, Sv, and Sr (Figure 5A). This model showed how three crops at Sr (MT, BR, and BG) still presented high amounts of dhurrin in their leaves and, generally, metabolomic features similar to the leaves at Sv. Among these three fields, BR and BG also showed a significant concentration of dhurrin in their grain, which is an established biomarker of low ripeness degree in sorghum.^30^ Conversely, in the case of MT, dhurrin was not found in the grain. These data remind that dhurrin is not only a biomarker of low ripeness degree but is also involved in other plant physiological processes and stress responses. In particular, the cyanogenic potential of sorghum is reported to greatly increase under abiotic stresses (drought, salinity, freezing, insufficient light, and nutrient deficiency) and herbivore and insect attack.^36^ In the case of MT, sorghum was probably subjected to overfertilization, as the very high nitrogen supply demonstrates (Table 2). In fact, it was proved that dhurrin biosynthetic enzymes are induced by nitrate availability.^14^

A high content of dhurrin is toxic to animals^37,38^ because of the release of HCN. Considering the fact that, together with grain, leaves and stems of sorghum are also generally used as forage, it is important to monitor and keep the level of this metabolite low in these organs also. This can be facilitated by the detection of environmental factors and practices correlated to the increase of this metabolite in sorghum.

**Figure 9:**
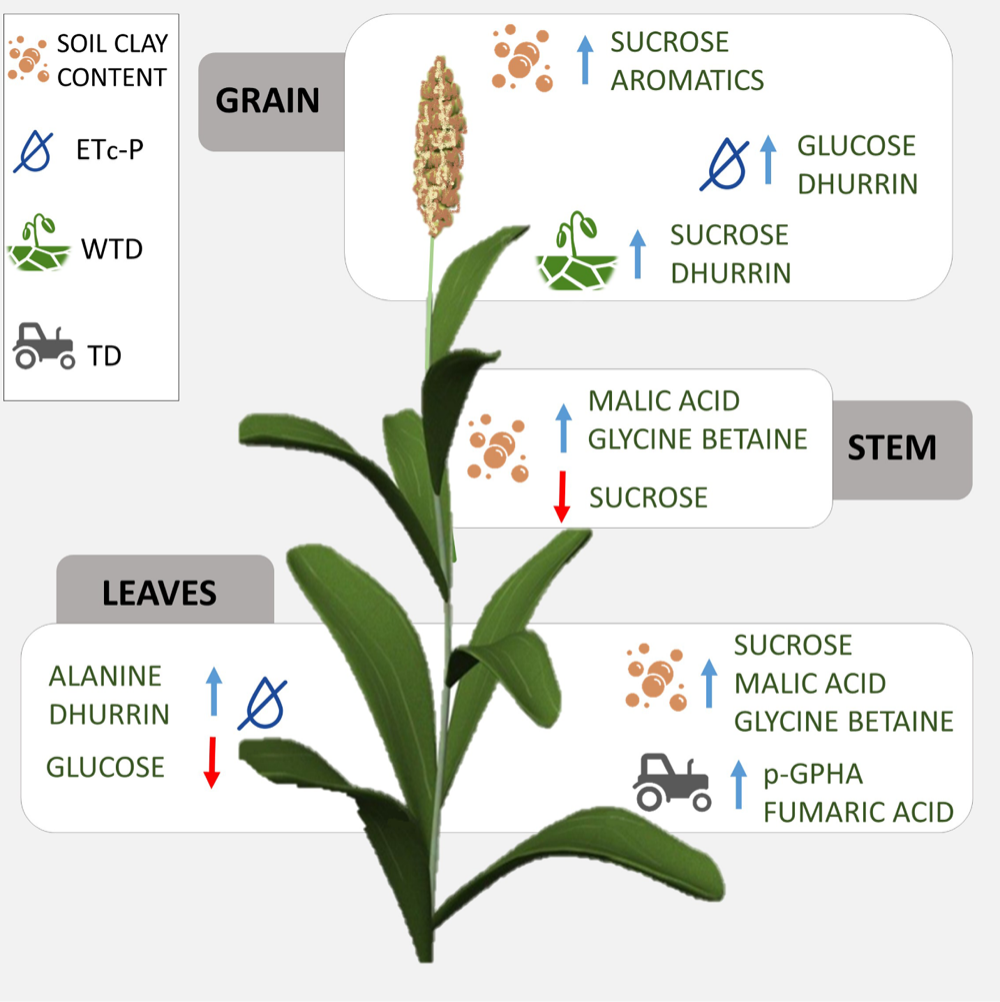
Schematic representation of the main agro-meteorological variables affecting the metabolite composition in sorghum at Sr. TD = tillage depth; WTD = depth of the shallow water table in July; ET_C_ – *P* = difference between crop evapotranspiration and precipitation. WTD and ET_C_ – *P* are related to water deficit.

In this work, metabolomic analysis was coupled with the assessment of crop and environmental parameters. This integrated approach allowed us to identify the most significant features related to sorghum metabolomic variations (Figure 9).

Several soil features were proved to be important for the diversification of sorghum metabolome at different plant stages (Figure S6). In particular, clay content was correlated to the metabolite variations in all plant organs at Sr, as shown by the *S*-plots of the developed OPLS models reported in Figure 7. Specifically, highest clay was correlated with an increase of sucrose in Sr leaves and a decrease of the same metabolite in Sr stems and grain. Clay content was also positively correlated to malic acid and glycine betaine (a marker of drought resistance in plants) in both the stems and leaves at Sr and to the increasing aromatic compounds in grain, whereas it did not affect the dhurrin content in any plant organ.

Another influencing parameter for Sr leaves was TD; indeed, it directly correlated to the increasing concentration of *p*-GPHA and fumaric acid, in addition to a slight increase in dhurrin concentration.

Difference in ET_C_ – *P* had an impact on the metabolomes of both leaves (at Sv and Sr) and grain, whereas WTD affected the grain selectively.

In leaves at Sr, increasing ET_C_ – *P* (which reflects the water deficit) was highly related to increasing dhurrin content, together with alanine. This effect is consistent with the findings of Wheeler *et al.*,^39^ who reported that a prolonged exposure to chronic water deficit induces higher cyanide potentials in sorghum plants. Our results also support the findings of Burke *et al.*,^40^ who demonstrated that the leaf dhurrin content is a quantitative measure of the level of pre- and post-flowering drought tolerance in sorghum.

The overall water deficit (increasing ET_C_ – *P* and decreasing WTD) also determined a slight increase of dhurrin in grain.

Carbohydrate levels have also been associated with water supply variation in sorghum varieties.^41^ According to our results, the sucrose level in grain was increased by higher WTD, whereas an opposite trend was shown for glucose. ET_C_ – *P* increase was correlated to a lower glucose accumulation in leaves at Sr, and it was associated to a higher glucose content in grain (Figure S6).

Regarding grain yield, the strong variation in sorghum behavior and final yield is not surprising even in a relatively small region under a similar course of weather in a specific year. Although sorghum has lower water requirement than maize,^42^ it is affected by long dry periods, as those experienced in the 12 fields in 2017. Together with the impact on the metabolome, this factor leads to a reduced yield, overall, and stronger variation among the cultivation sites; the almost 1:4 yield ratio between the worst and best cases (Table 1) supports this point. However, grain yield, which is the ultimate goal for the farmer, was neither significantly correlated with the two parameters (ET_C_ – *P* and ET_C_/*P*) (Table 2), expressing water deficit in the whole crop cycle, nor with any of the four stages (initial, development, mid, and late) in which the crop cycle is subdivided for computing ET_C_^19^ (data not shown). This means that other factors must have played a major role, as the occurrence of a shallow water table contributing to sorghum water supply. Interactions with ET_C_ – *P* and ET_C_/*P* are, nevertheless, not excluded.

Although commercial sorghum grain is allowed to contain a low quantity of dhurrin, a better quality grain, especially for human consumption, is out of doubt dhurrin-free.

Our study highlighted that three sorghum fields (BG, BR, and SH) yielded grain still containing traces of dhurrin; among them, SH also showed the lowest antioxidant activity. Dhurrin content in sorghum grain generally decreases with ripening. Notably, in the case of BR and BG, the delay in ripening was prospected at Ss by their metabolomic profiles having a high content of dhurrin. Farmers commonly harvest sorghum under the influence of the weather or other crop management urgencies. This practice may lead to premature harvesting, which, according to our results, determines lower quality grain as in the case of BR and BG.

To conclude, metabolomics combined with the surveyed agronomic parameters through multivariate data analysis turned out to be a valid tool to develop smart agriculture practices, enabling predictions on sorghum development trend since the seedling stage. This could provide information on the most appropriate agricultural practices since the early stage of crop management, in order to obtain better grain quality, together with residual plant organs (leaves and stems) better suited for feeding livestock. The models obtained represent a useful starting point for implementing further studies on sorghum, which are ongoing.

## Supporting Information Available

NMR references and MS data for dhurrin and chlorogenic acid; model of multivariate data analysis performed and associated parameters; diagnostic signals in the ^1^H NMR profile of the detected metabolites; chemical shifts, splitting pattern, and coupling constants; VIP of the most variated metabolites resulting from PLS-DA and OPLS-DA; loading plots of ^1^H NMR-based PCA model for seedlings; loading plots of ^1^H NMR-based PCA model for sorghum at Sv; loading plots of ^1^H NMR-based PCA model for sorghum at Sr; ^1^H NMR-based PCA of sorghum stems at Sv, OPLS-DA score scatterplot comparing stems and leaves at Sv, PCA score scatterplot of stems at Sr, and OPLS-DA score scatterplot of stems and leaves at Sr; loading plots of ^1^H NMR-based PCA model for grain; and *S*-plots obtained from different OPLS models using different *y* variables (*y* = TD, ET_C_ – *P*, H-ini, OC, and silt) (PDF)

## Abbreviations

BCB: β-carotene bleaching assay
DW: dry weight
ET_0_: reference evapotranspiration
ET_C_: crop evapotranspiration
ET_C_ – *P*: difference between crop evapotranspiration and precipitations
H-ini: soil humidity at the beginning of the sorghum growth season
H-mid: soil humidity at the middle of the sorghum growth season
H-late: soil humidity at the end of the sorghum growth season
OC: organic carbon content at 0–30 cm depth
*p*-GPA: *p*-glycosyloxyphenyl-acetic acid
*p*-GPHA: *p*-glucosyloxy-2-hydroxyphenylacetic acid
*p*-HMN: *p*-hydroxymandelonitrile
*p*-HBA: *p*-hydroxybenzaldehyde
Sr: ripening stage
Ss: seedling stage
Sv: advanced vegetative stage
TD: tillage depth
WTD: depth of the shallow water table in July
