# Supplementary material for: Metabolomic Study of Sorghum (Sorghum bicolor) to Interpret Plant Behavior under Variable Field Conditions in View of Smart Agriculture Applications
Source: J Agric Food Chem. 2021 Jan 18;69(3):1132–45. doi: 10.1021/acs.jafc.0c06533 (PMC8769377; doi:10.1021/acs.jafc.0c06533)
Supplement: Supplementary file 1 — jf0c06533_si_001.pdf [file jf0c06533_si_001.pdf]

Supporting Information

Metabolomic study of sorghum (*S. bicolor*) to interpret plant behavior under variable field conditions in view of smart agriculture applications

Manuela Mandrone[a*§], Ilaria Chiocchio[a§], Lorenzo Barbanti[b], Paola Tomasi[a], Massimo Tacchini[c], Ferruccio Poli[a]

[a] Department of Pharmacy and Biotechnology, University of Bologna, Via Irnerio, 42, 40126 Bologna, Italy

[b] Department of Agricultural and Food Sciences, Viale Fanìn 44, 40127 Bologna, Italy

[c] Department of Life Sciences and Biotechnology (SVeB), University of Ferrara, Piazzale Luciano Chiappini 3, I-44123, Ferrara, Italy

[§]These authors equally contributed to write this work

***Correspondence**

Dr. Manuela Mandrone, University of Bologna, Department of Pharmacy and Biotechnology, Via Irnerio 42, 40126 Bologna, Italy

E-mail: manuela.mandrone2@unibo.it Phone: +390512091294; Fax +39051242576

Table S1. **Model of multivariate data analysis performed and associated parameters.** For OPLS models the *y* variable (*y* var.) used is reported, and for discriminant analyses the classes used are given (Class ID). Goodness of fit (R^2Y(cum), (R^2X(cum)) and goodness of prediction (Q^2(cum)), together with the parameters given by the permutation test (R^2 perm. and Q^2 perm.) were evaluated to establish if the developed supervised models were interpretable. Models resulting not interpretable are not reported in this table.

Model	*y* var.	Class ID	Comp. numb.	R^2X(cum)	R^2Y(cum)	Q^2(cum)	R^2 prem.	Q^2 perm.	*x* var. (^{1}H NMR profile)
**OPLS**	OC	-	5	0.909	0.977	0.644	0.977	0.664	stems Sv
**OPLS**	Silt	-	9	0.985	1	0.899	1	0.899	stems Sv
**OPLS**	H-ini	-	2	0.778	0.814	0.634	0.814	0.634	stems Sv
**OPLS**	Clay	-	3	0.739	0.84	0.521	0.84	0.521	stems Sr
**OPLS**	Silt	-	3	0.683	0.808	0.233	0.808	0.233	stems Sr
**OPLS**	TD	-	10	0.989	1	0.847	0.989	0.847	leaves Sr
**OPLS**	OC	-	3	0.765	0.825	0.364	0.825	0.364	leaves Sr
**OPLS**	Clay	-	7	0.944	0.999	0.861	0.999	0.861	leaves Sr
**OPLS**	Silt	-	2	0.685	0.51	0.171	0.51	0.171	leaves Sr
**OPLS**	ET_C-P	-	5	0.884	0.993	0.774	0.993	0.774	leaves Sr
**OPLS**	H-mid	-	8	0.969	0.999	0.82	0.999	0.82	leaves Sr
**OPLS**	H-late	-	8	0.965	0.999	0.812	0.999	0.812	leaves Sr
**OPLS-DA**	-	Growth stage	2	0.808	0.96	0.947	0.96	0.947	stems Sr and leaves Sr
**OPLS-DA**	-	Growth stage	2	0.555	0.949	0.902	0.949	0.902	stems Sv and leaves Sv
**PCA**	-	-	2	0.764		0.656	-	-	aerial parts Ss
**PCA**	-	-	3	0.863		0.227	-	-	leaves Sv
**PCA**	-	-	2	0.788		0.616	-	-	stems Sv
**PCA**	-	-	2	0.698		0.453	-	-	leaves Sr
**PCA**	-	-	2	0.705		0.49	-	-	stems Sr
**OPLS-DA**	-	Growth stage	2	0.853	0.94	0.898	0.94	0.898	stems Sv and stems Sr
**PCA**	-	-	2	0.668	0.34	-	-	-	grain
**OPLS**	dhurrin concen tration	-	2	0.613	0.903	0.682	0.902	0.682	grain
**OPLS-DA**	ET_C-P	-	8	0.925	1	0.821	0.99	0.82	grain
**OPLS-DA**	WTD	-	7	0.917	0.999	0.818	0.999	0.81	grain
**OPLS-DA**	Clay	-	8	0.98	1	0.553	0.999	0.553	grain
**PLS-DA**	-	Growth stage	3	0.846	0.88	0.853	0.916	0.893	Ss, Sr and Sv leaves

Table S2. Diagnostic signals in ^{1}H NMR profile of the detected metabolites. Chemical shifts (δ), splitting pattern and coupling constants (Hz) are reported.

Metabolite	diagnostic signals in ^{1}H NMR profiles
alanine	1.49 (d, J = 7.2 Hz)
acetate	1.95 (s)
p-hydroxybenzaldehyde	9.74 (s), 7.85 (d, J = 8.6 Hz), 7.01 (d, J = 8.6 Hz)
dhurrin	7.51 (d, J = 8.6 Hz), δ 6.99 (d, J = 8.6 Hz), δ 5.96 (s), δ 4.77 (d, J = 7.8 Hz)
fumaric acid	6.66 (s)
α-glucose	5.19 (d, J = 3.8 Hz)
β-glucose	4.58 (d, J =7.8 Hz)
malic acid	4.28 (dd, J = 9.2, 3.4 Hz), 2.70 (dd, J = 15.7, 3.5 Hz)
sucrose	5.41 (d, J = 3.8 Hz), δ 4.17 (d, J = 8.6 Hz)
p-GPHA	7.39 (d, J = 8.6 Hz), 7.09 (d, J = 8.6 Hz), 5.02 (d, J = 7.6 Hz), 4.89 (s)
valine	1.00 (d, J = 7.00 Hz), 1.05 (d, J = 7.00 Hz)
threonine	1.34 (d, J = 6.48 Hz)
aspartate	2.95 (dd, J = 16.9, 4.1 Hz), δ 2.81 (dd, J = 16.9, 8.2 Hz)
chlorogenic acid	7.55 (d, J = 15.9 Hz), 7.13 (d, J = 1.9 Hz), 7.07 (dd, J = 8.2, 1.9 Hz), 6.89 (d, J = 8.2 Hz), 6.30 (d, J = 15.9 Hz) 5.29 (m), 4.26 (m), 3.88 (dd, J = 8.8, 3.3 Hz), 2.23 (m), 2.10 (m)
glycine betaine	3.28 (s)
succinate	2.34 (s)
trigonelline	9.14 (s); 8.87 (m)

Table S3. Variable influence on projection (VIP) of the most variated metabolites resulting from PLS-DA (performed on leaves at Ss, Sv and Sr) and OPLS-DA (performed on stems at Sv and Sr).

Metabolite	Bucket region (δ)	VIP of PLS-DA	VIP of OPLS-DA
glycin betaine	3.23571 - 3.27571	2.92217	2.27932
sucrose	5.39571 - 5.43571	2.43905	1.67366
dhurrin	6.91571 - 6.95571	2.24026	0.746258
malic acid	4.27571 - 4.31571	2.07805	0.863811
chlorogenic acid	3.87571 - 3.91571	1.9133	-
p-GPHA	7.07571 - 7.11571	1.56182	-
p-HBA	6.99571 - 7.03571	1.46096	0.364999
threonine	1.31571 - 1.35571	1.15579	0.532396

β-glucose	4.55571 - 4.59571	0.94673	2.6686
α-glucose	5.15571 - 5.19571	0.808429	2.06927
aspartic acid	2.79571 - 2.83571	0.790976	0.902605
alanine	1.47571 - 1.51571	0.764678	0.612495
fumaric acid	6.63571 - 6.67571	0.663863	0.683252
succinic acid	2.36699 - 2.40699	-	0.863811
valine	1.04699 - 1.08699	-	0.625918
acetic acid	1.96699 - 2.00699	-	0.601574

Paragraph S1. Dhurrin and chlorogenic acid NMR references and MS data

Dhurrin ^{1}H NMR (CD_3OD, 600 MHz): δ 7.51 (d, 2, J = 8.6 Hz, H-4, H-8), 6.99 (d, 2, J = 8.6 Hz, H-5, H-7), 5.96 (s, 1, H-2), 4.77 (d, 1, J = 7.8 Hz, H-1'), 3.95 (dd, 1, J = 12.5, 2.3 Hz, H-6'b), 3.76 (dd, 1, J = 12.5; 5.8 Hz, H-6'a), 3.54 (t, 1, J = 9.2 Hz, H-3'), 3.53 (qd, 1, J = 9.9, 5.9, 2.3 Hz, H-5'), 3.43 (t, 1, J = 9.5 Hz, H-4'), 3.34 (dd, 1, J = 8.3, 3.8 Hz, H-2'); ^{13}C NMR (CD_3OD, 150 MHz): δ 157.3 (COH, C-6), 130.00 (CH, C-4, C-8), 124.21 (C, C-3), 117.85 (CN, C-1), 115.98 (CH, C-5, C-7), 100.13 (COH, C-1'), 75.81 (CH, C-5'), 75.46 (CH, C-3'), 72.76 (CH, C-2'), 69.25 (CH, C-4'), 68.21 (C, C-2), 60.39 (CH_2, C-6'a, C-6'b).

Chlorogenic acid ^{1}H NMR (CD_3OD, 600 MHz): δ 7.55 (d, 1, J = 15.9 Hz, H-7'), 7.13 (d, 1, J = 1.9 Hz, H-2'), 7.07 (dd, 1, J = 8.2, 1.9 Hz, H-6'), 6.89 (d, 1, J = 8.2 Hz, H-5'), 6.30 (d, 1, J = 15.9 Hz, H-8') 5.29 (m, 1, H-3), 4.26 (m, 1, H-5), 3.88 (dd, 1, J = 8.8, 3.3 Hz, H-4), 2.23 (m, 2, H-2a, H-2b), 2.10 (m, 2, H-6a, H-6b) ^{13}C NMR (CD_3OD, 150 MHz): δ 168.85 (COO, C-9'), 146.16 (CH, C-7'), 114.40 (CH, C-8'), 122.80 (CH, C-6'), 115.17 (CH, C-2'), 116.25 (CH, C-5'), 146.77 (CO, C-4'), 144.34 (CO, C-3'), 137.91 (C, C-1'), 70.85 (CO, C-3), 71.84 (CO, C-4), 69.71 (CO, C-5), 37.04 (CH, C-2), 36.70 (CH, C-6), 75.57 (CO, C-1). Positive ESI-MS m/z: 393 $[M + K]^+$, 377 $[M + K]^+$, calculated as 354.10 for $C_{16}H_{18}O_9$. Negative ESI-MS m/z: 353 $[M – H]^-$.

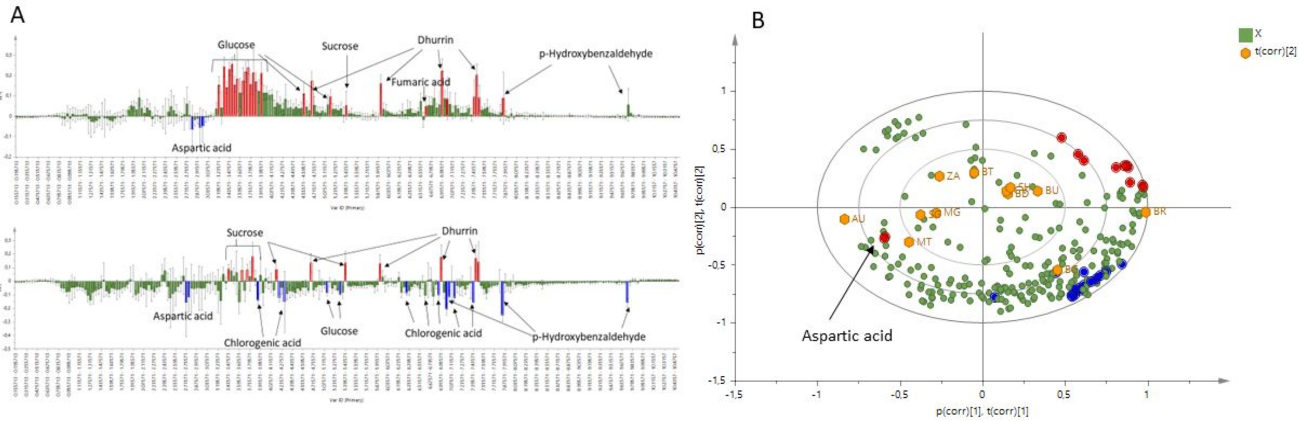

Fig. S1. Loadings plot of ^{1}H NMR-based PCA of sorghum at Ss. (A) Loadings plots of PC1 (up) and PC2 (down). **(B)** Biplot.

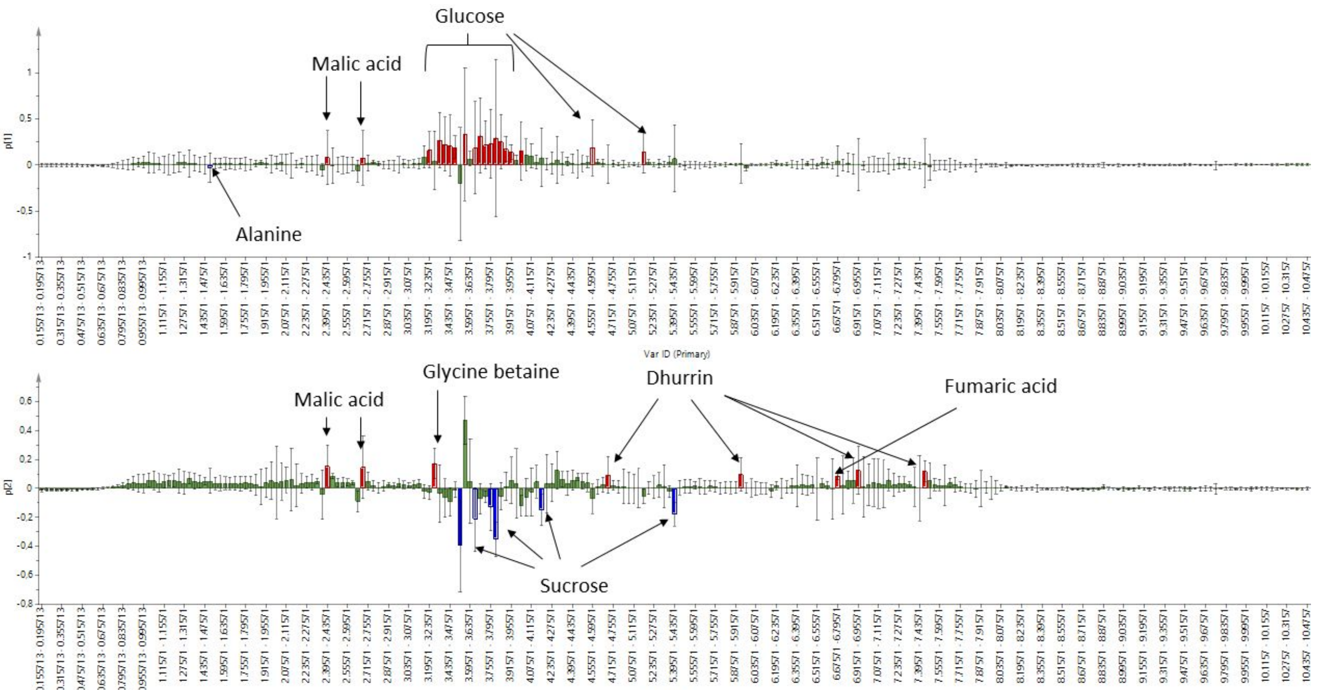

Fig. S2. ^{1}H NMR-based PCA of sorghum at Sv. Loadings plots of PC1 (up) and PC2 (down).

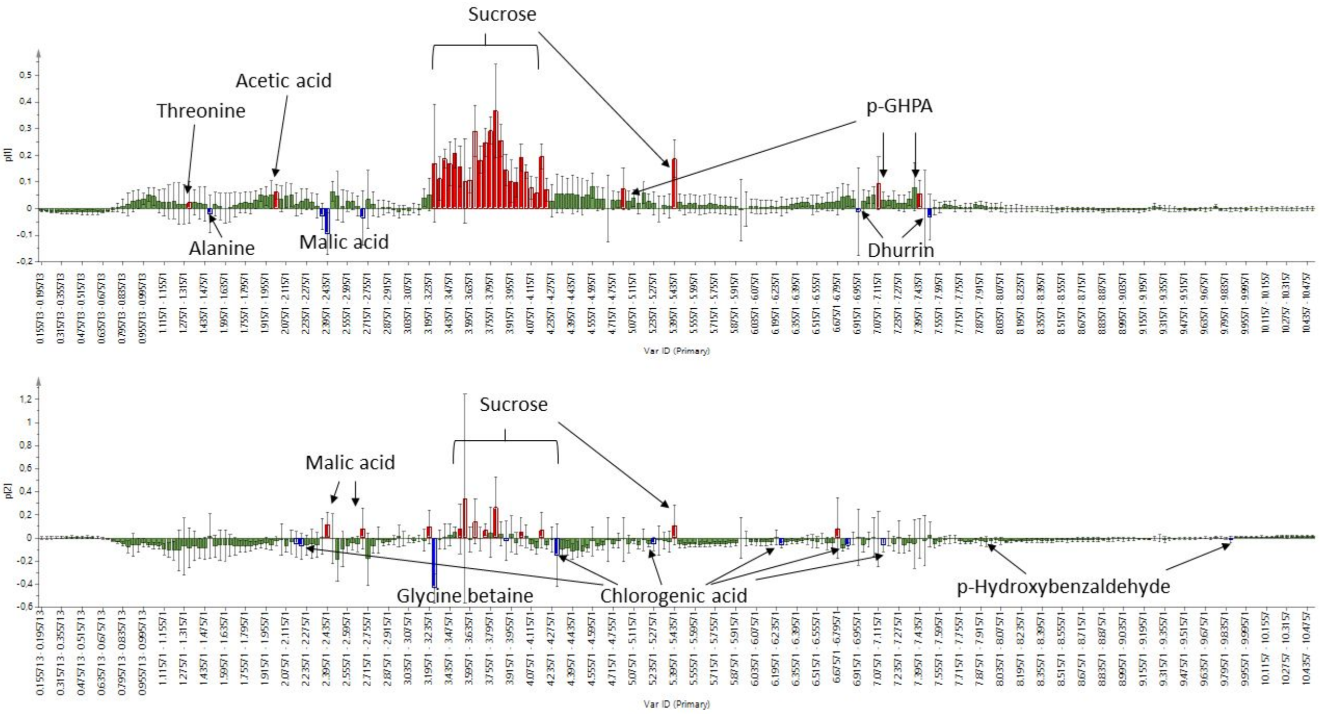

Fig. S3. ^{1}H NMR-based PCA of sorghum at Sr. Loadings plots of PC1 (up) and PC2 (down).

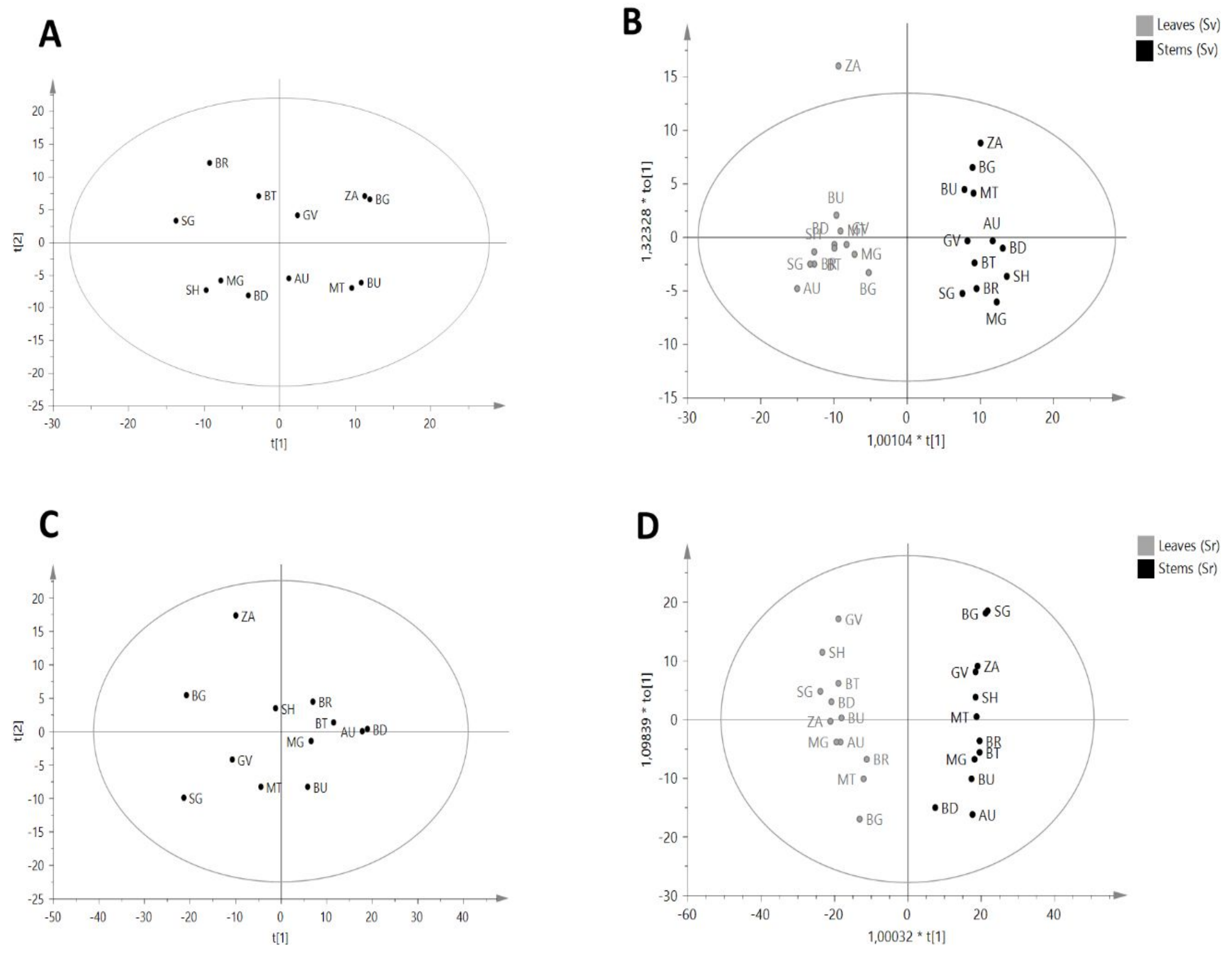

Fig. S4. ^{1}H NMR-based (A) PCA of sorghum stems at Sv. Fumaric acid increases along the negative side of t[1] while dhurrin increases on the positive side of t[1]; glucose and aspartate increase on the positive side of t[2], while sucrose increases on the negative side of t[2]. **(B) OPLS-DA score scatter plot comparing stems and leaves at Sv.** *p* (CV-ANOVA)=2.44 x 10^{-9}; F (CV-ANOVA)=43.85 **(C) PCA score scatter plot of stems at Sr. (D) OPLS-DA score scatter plot of stems and leaves at Sr.** *p* (CV-ANOVA)=7.92 x 10^{-12}; F (CV-ANOVA)=84.44

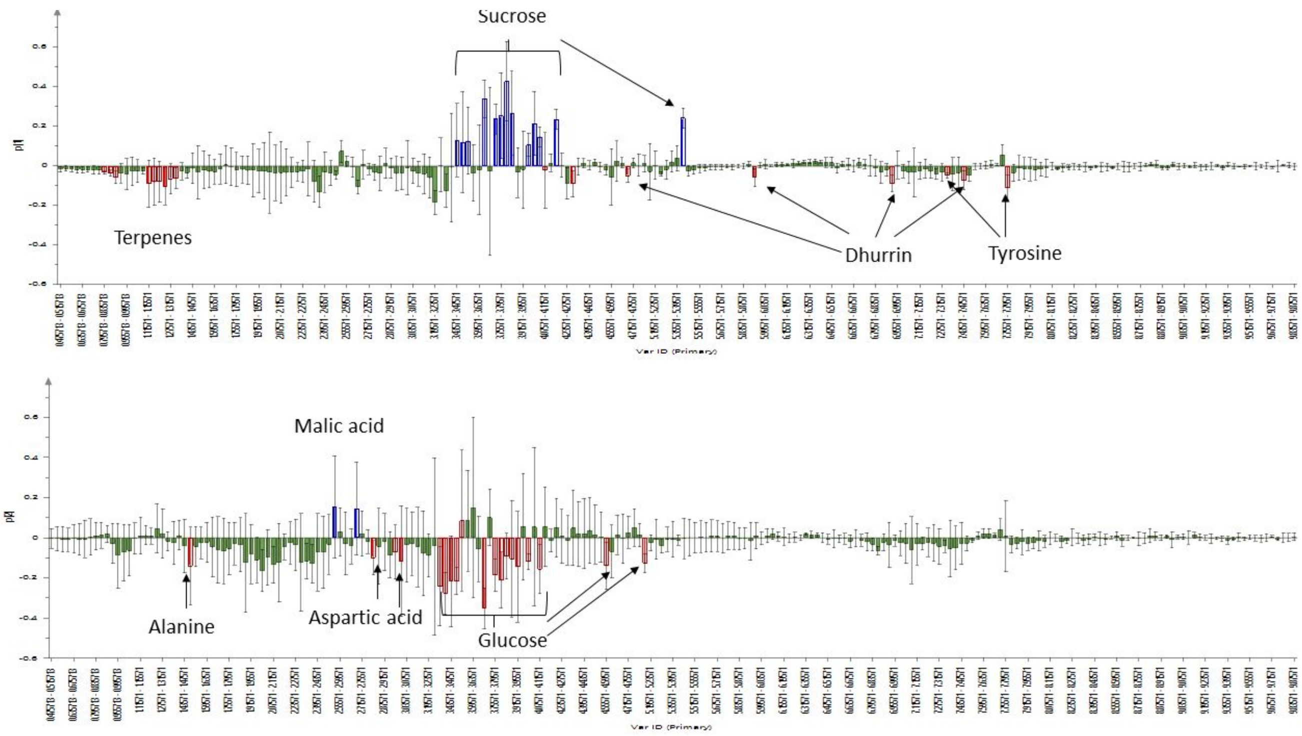

Fig. S5. ¹H NMR-based PCA of grain. Loadings plots of PC1 (up) and PC2 (down).

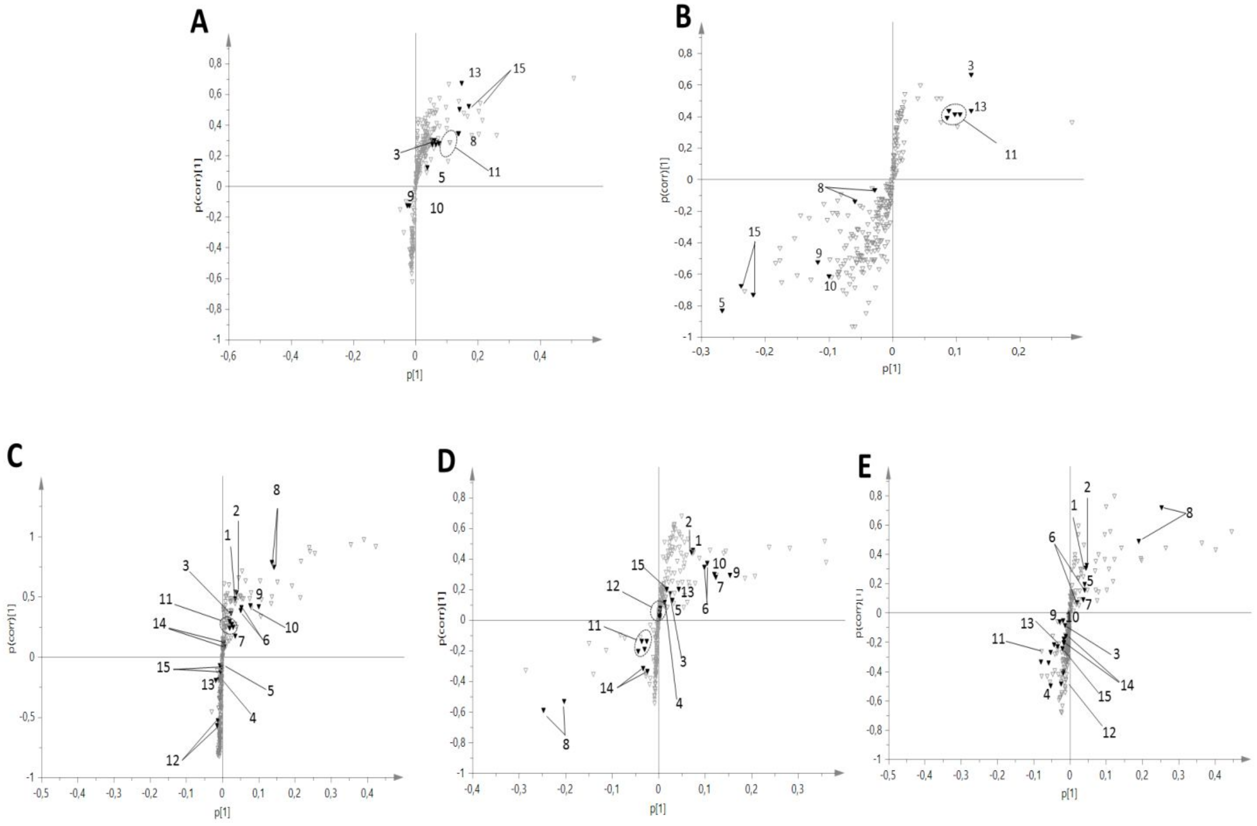

Fig. S6. *S*-plot obtained from different OPLS models using different *y* variable. (A) Leaves at Sr correlated to *y*= TD, **(B)** Leaves at Sr correlated to *y*= Etc-P. **(C)** Stems at Sv correlated to *y*=H-ini, **(D)** Stems at Sv correlated to *y*=OC, **(E)** Stems at Sv correlated to *y*=Silt**.** 1=valine, 2=threonine, 3=alanine, 4=acetate, 5=malic acid, 6=aspartic acid, 7=glycine betaine, 8=sucrose, 9=β-glucose, 10=α-glucose, 11=dhurrin, 12=chlorogenic acid, 13=fumaric acid, 14=p-OH-benzaldehyde, 15=p-GHPA, 16=lipids, 17= aromatics, 18=trigonelline.
